# *In Situ* Overexpression of Matricellular Mechanical Proteins Demands Functional Immune Signature and Mitigates Non-Small Cell Lung Cancer Progression

**DOI:** 10.3389/fimmu.2021.714230

**Published:** 2021-08-16

**Authors:** Lygia Bertalha Yaegashi, Camila Machado Baldavira, Tabatha Gutierrez Prieto, Juliana Machado-Rugolo, Ana Paula Pereira Velosa, Lizandre Keren Ramos da Silveira, Aline Assato, Alexandre Muxfeldt Ab’Saber, Roberto Falzoni, Teresa Takagaki, Pedro Leme Silva, Walcy Rosolia Teodoro, Vera Luiza Capelozzi

**Affiliations:** ^1^Department of Pathology, University of São Paulo Medical School, São Paulo, Brazil; ^2^Health Technology Assessment Center (NATS), Clinical Hospital (HCFMB), Medical School of São Paulo State University (UNESP), Botucatu, Brazil; ^3^Rheumatology Division of the Hospital das Clinicas, University of São Paulo Medical School, São Paulo, Brazil; ^4^Division of Pneumology, Instituto do Coração (Incor), University of São Paulo Medical School (USP), São Paulo, Brazil; ^5^Laboratory of Pulmonary Investigation, Carlos Chagas Filho Biophysics Institute, Federal University of Rio de Janeiro, Centro de Ciências da Saúde, Rio de Janeiro, Brazil; ^6^National Institute of Science and Technology for Regenerative Medicine, Rio de Janeiro, Brazil

**Keywords:** lung cancer, immune cells, collagen, cancer-associated fibroblasts, immunofluorescence, immunohistochemistry, non-small cell lung cancer

## Abstract

Non-small cell lung carcinoma (NSCLC) is a complex cancer biome composed of malignant cells embedded in a sophisticated tumor microenvironment (TME) combined with different initiating cell types, including immune cells and cancer-associated fibroblasts (CAFs), and extracellular matrix (ECM) proteins. However, little is known about these tumors’ immune-matricellular relationship as functional and mechanical barriers. This study investigated 120 patients with NSCLC to describe the immune-matricellular phenotypes of their TME and their relationship with malignant cells. Immunohistochemistry (IHC) was performed to characterize immune checkpoints (PD-L1, LAG-3, CTLA-4+, VISTA 1), T cells (CD3+), cytotoxic T cells (CD8^+^, Granzyme B), macrophages (CD68+), regulatory T cells (FOXP3+, CD4+), natural killer cells (CD57+), and B lymphocytes (CD20+), whereas CAFs and collagen types I, III, and V were characterized by immunofluorescence (IF). We observed two distinct functional immune-cellular barriers—the first of which showed proximity between malignant cells and cytotoxic T cells, and the second of which showed distant proximity between non-cohesive nests of malignant cells and regulatory T cells. We also identified three tumor-associated matricellular barriers: the first, with a localized increase in CAFs and a low deposition of Col V, the second with increased CAFs, Col III and Col I fibers, and the third with a high amount of Col fibers and CAFs bundled and aligned perpendicularly to the tumor border. The Cox regression analysis was designed in two steps. First, we investigated the relationship between the immune-matricellular components and tumor pathological stage (I, II, and IIIA), and better survival rates were seen in patients whose tumors expressed collagen type III > 24.89 fibers/mm². Then, we included patients who had progressed to pathological stage IV and found an association between poor survival and tumor VISTA 1 expression > 52.86 cells/mm² and CD3+ ≤ 278.5 cells/mm². We thus concluded that differential patterns in the distribution of immune-matricellular phenotypes in the TME of NSCLC patients could be used in translational studies to predict new treatment strategies and improve patient outcome. These data raise the possibility that proteins with mechanical barrier function in NSCLC may be used by cancer cells to protect them from immune cell infiltration and immune-mediated destruction, which can otherwise be targeted effectively with immunotherapy or collagen therapy.

## Introduction

The progression and prognosis of non-small cell lung carcinomas (NSCLC) remain a problem for both oncologists and patients. The median survival among patients who receive targeted therapy is poor (approximately 8 months), and only 15% of patients are alive after 5 years (1). Furthermore, nearly 50% of NSCLC patients develop distant metastases or relapse, one third develop brain metastases, and several cases of occult spread beyond the lung have been described even when the tumor seemed to have been completely resected (2). Thus, additional strategies are still being sought to improve this scenario.

NSCLC is a complex cancer biome composed of malignant cells embedded in a sophisticated tumor microenvironment (TME) with different and combined initiating cell types, including CAFs, and extracellular matrix (ECM) proteins. The TME is regulated by continuous epithelial-to-mesenchymal-ECM interactions, which are now recognized as key actors in the theatrical scenario for progression of primary lung carcinoma (3–6). In the ECM, a variety of collagen proteins provide the framework for parenchymal and stroma alignment, working as a mechanical barrier that regulates cancer cell growth and motility (7), whereas immune checkpoints, macrophages, T cells, and natural killer (NK) cells represent the functional immune barrier.

T lymphocytes encompass 80% of tumor-infiltrating lymphocytes (TILs) (8), and among them, CD8^+^ cytotoxic lymphocytes represent the effector arm of adaptive immunity anti-tumoral cells that promote the delay in cell proliferation and growth rates. Investigations that examined the association between CD8^+^ TILs and prognosis in NSCLC are controversial (9, 10). Moreover, a study including a large casuistic of patients with NSCLC showed an association of CD8^+^ and longer survival only in squamous cell carcinoma (10), in opposite to the findings of Wakabayashi and colleagues (9). In other reports, CD3 with simultaneous high infiltration of CD4+/CD8^+^ correlated with longer survival (11, 12). Equally, statements have been made for high CD4/CD8 and CD20 lymphocyte infiltration in the stroma (9, 10). High FOXP3 or density of NK cells also have been reported to correlate with the prognosis of recurrence in cancer (13).

Most of those studies, looking for biomarkers that modulate the risk of cancer progression and specific death, have primarily focused on tumor epithelial compartment (14, 15) and not on their relationship with collagen and CAFs in tumor stroma for invasion more lethal, and more therapeutically relevant for metastatic lesion. Therefore, there is a pressing need for a pathway that helps oncologists identify which patients are most expected to relapse or develop metastases after surgery (16). Additional molecular categorization of the tumor landscape could bring the opportunity to identify novel factors and matched molecular targets that control disease progression and thus lead to the conception of innovative therapeutic strategies (17).

In order to address these gaps in the literature, we evaluated the relationship between those known markers of the immunological journey in NSCLC and matricellular proteins, most of them involved in functional and mechanical molecular barriers and cell block motility that support NSCLC invasion and metastases in localized surgically resected primary tumor. Overall, overexpression of matricellular mechanical barrier proteins was associated with two distinct patient populations with lack immune signature and immune-suppressive barrier in these cancers, but with different prognostic implications for each. These data raise the possibility that proteins with mechanical barrier function in lung tissue may be used by cancer cells to protect them from immune cell infiltration and immune-mediated destruction.

## Materials and Methods

### Study Cohort

We conducted a retrospective, longitudinal, single-center study which included a consecutive series of 120 NSCLC patients who underwent curative surgical resection between 1995 and 2018 at the Thoracic Surgery Unit of Clinicas Hospital, at the Heart Institute (InCor), and at the São Paulo Cancer Institute (ICESP) of the University of São Paulo Medical School. We included in the study chemo-naive patients with histologically diagnosed NSCLC stages I, II, or IIIA whose thoracic surgery had produced adequate tissue specimens. Patients treated with neoadjuvant chemotherapy and/or radiotherapy, submitted to palliative surgical procedure, or with inadequate paraffin embedded formalin fixed specimens were excluded.

Clinicopathological data were collected and managed using REDCap electronic data capture tools hosted at ICESP and included gender, age, tobacco history, histology, and disease stage, as described in the Eighth Edition of the Union for International Cancer Control (UICC) TNM Classification of Malignant Tumors (18). We also collected information on subsequent systemic or locoregional treatments, eventual relapse, and death. Patients were followed up on an outpatient basis through monthly visits to the oncologist. Brain, chest, and abdomen CT scans were performed every 6 months for the first 5 years and every year thereafter. Overall survival (OS) served as the primary outcome and was defined as the interval from surgery to death or last contact.

This study was conducted according to the current rules of Good Clinical Practice and principles of the Helsinki declaration. The Internal Ethics Committees of all participating institutions approved this study protocol under number 2.394.595.

### Sample Selection Criteria

In the current study, to achieve our objectives, consecutive samples of NSCLC from our patients were selected to characterize immune checkpoints (PD-L1, LAG-3, CTLA-4+, VISTA 1), T cells (CD3+), cytotoxic T cells (CD8^+^, Granzyme B), macrophages (CD68+), regulatory T cells (FOXP3+, CD4+), NK cells (CD57+), and B lymphocytes (CD20+), as well as matricellular proteins (cancer-associated fibroblasts, collagen types I, III, and V). Formalin-fixed paraffin-embedded specimens of NSCLC resected were extracted from the archive of the Department of Pathology, Sao Paulo University. A cohort of all patients was identified for TMA construction. Diagnoses were made according to the recommendations of the World Health Organization classification for lung cancer 2015 (19) by an experienced lung pathologist. Twenty-five percent (N = 30) of our cases were poorly differentiated carcinomas, so immunohistochemistry was performed, and the final classification included adenocarcinoma (N = 73, 60%), squamous cell carcinoma (N = 24, 20%), and large cell carcinoma (N = 7, 6%).

**Figure 1:**
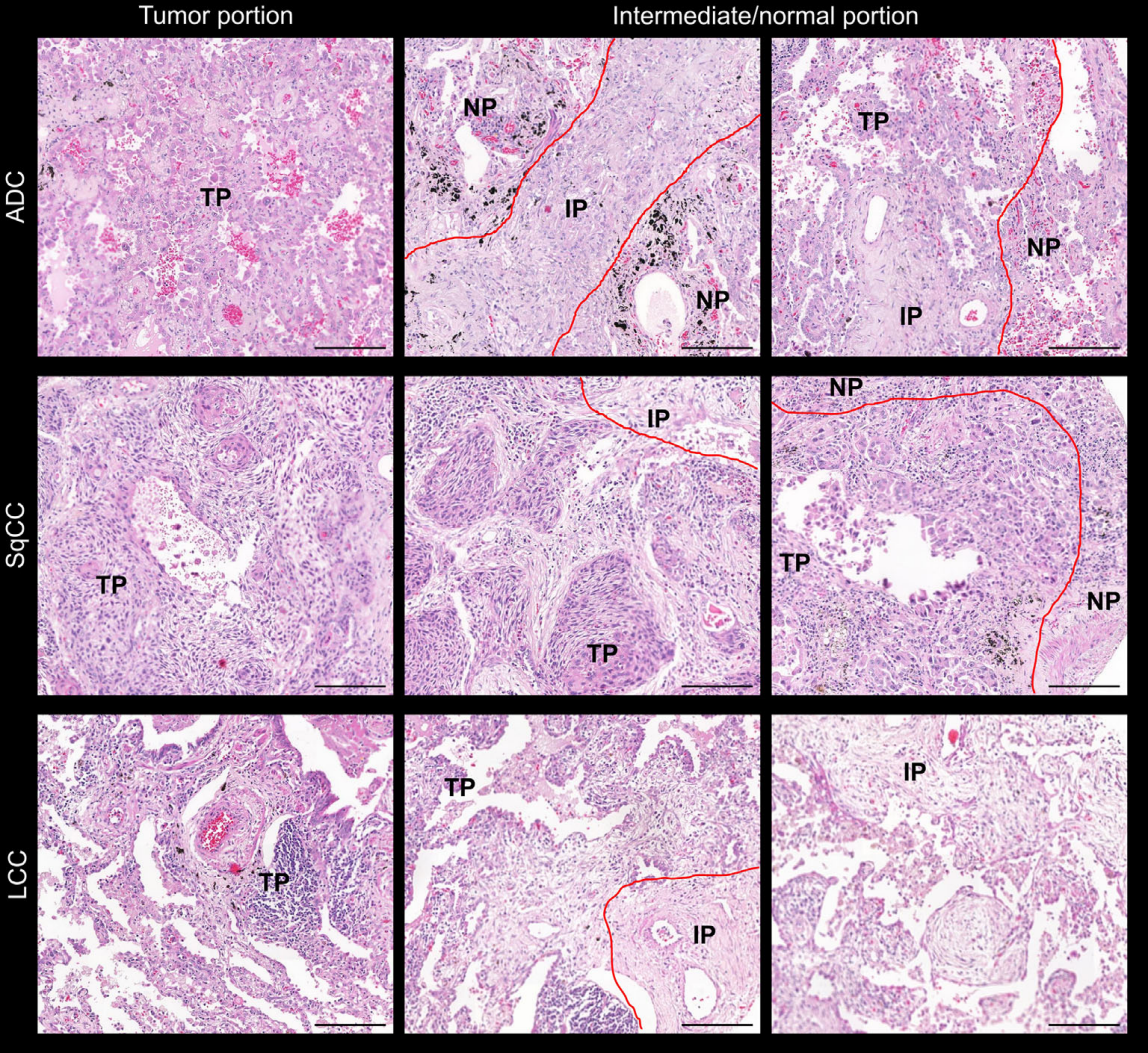
Microphotographs of representative examples of the TMAs stained by H&E in lung ADC, SCC, and LCC specimens. Lung tissue was divided into three comparments: normal tissue, tumor tissue, and intermediate (red lines). ADC, adenocarcinoma; LCC, large cell carcinoma; SqCC, squamous cell carcinoma; TP, tumor portion; IP, intermediate portion; NP, normal portion. Scale bar: 1 cm.

Prior to TMA construction, hematoxylin and eosin (H&E)-stained slide of each block was analyzed in order to ensure that TME would be represented by portions of normal tissue, intermediate, and tumor tissue as shown in **Figure 1**. Three TMA cores were available, and the TMA sections were prepared using three 1.0-mm tissue cores obtained from the three portions of the tumor, as described previously (20). Normal liver and kidney tissues were used for control and slide orientation purposes.

### Immunohistochemistry

To characterize and standardize the immune cell population, immunostaining was first tested on whole tissue sections and TMAs. In this step, four-micron-thick sequential whole tissue sections and TMA sections were stained with immunoperoxidase. For the immune checkpoints, the staining was performed with antibodies against programmed death ligand 1 (PD-L1; Ventana^®^, SP263), lymphocyte activating gene 3 (LAG-3; 1:100; Cell Signaling Technology), cytotoxic T-lymphocyte associated protein 4 (CTLA-4; 1:100; Santa Cruz), and V-domain Ig Suppressor of T cell Activation (VISTA 1; 1:400; Cell Signaling Technology). In parallel, we used antibodies against inflammatory markers: T cells (CD3, 1:15000; Dako), cytotoxic T cells (CD8, 1:400; Dako; and Granzyme B, 1:50; Biocare), NK cells (CD57, 1:100; Biocare), regulatory T cells (CD4, 1:100; Cell Signaling Technology; and Fork head box protein P3 - FOXP3, 1:100; Abcam), B lymphocytes (CD20, 1:2000; Dako), and macrophages (CD68, 1:5000; Dako). Briefly, the evaluation of immune cells and immune checkpoints was carried out in tumor epithelial and tumor stroma compartments, within the borders of the invasive tumor and in areas within the tumor, considering both intraepithelial and tumoral stromal areas.

### Immunofluorescence

The matricellular components including CAFs, collagen I, collagen III, and collagen V were characterized by immunoperoxidase followed by immunofluorescence. To perform the immunostaining, lung sections (3–4 μm) were mounted on 3-aminopropyltriethoxysilane (Sigma Chemical Co., St. Louis, MO, USA), dewaxed in xylol, hydrated in graded ethanol, and exposed to a 0.3% hydrogen peroxide and formic acid solution to inhibit endogenous peroxidase activity. Antigen retrieval was accomplished using a citrate buffer solution at pH 9.0 and heated in a Pascal pressure cooker (125°C for 1 min). Nonspecific sites were blocked with 5% bovine serum albumin (BSA) in phosphate buffer saline (PBS) for 30 min at room temperature. The specimens were incubated overnight at 4°C with rabbit polyclonal anti-human collagen type I (Col I; 1:700; Rockland, Inc., Limerick, Pennsylvania), anti-human collagen type III (Col III; 1:200; Rockland., Limerick, Pennsylvania), and anti-human collagen type V (Col V; 1:1000; Rockland Inc., Limerick, Pennsylvania) antibodies diluted in 0.01% BSA associated which monoclonal anti-human α-smooth muscle actin (AML;1:100; Dako Cytomation - clone 1A4 - cod. M0851 - Denmark) and monoclonal anti-human Vimentin (Vim; 1:100; Novocastra-clone V9- cod. NCL-L-VIM-09 Newcastle – UK) to characterize CAFs. These lung sections were then washed in PBS with Tween 20 at 0.05% and incubated for 60 min at room temperature with Alexa 488-conjugated goat anti-mouse IgG (1:200, Invitrogen, Eugene, OR, USA) and Alexa 488-conjugated goat anti-rabbit IgG (1:200, Invitrogen, Eugene, OR, USA). For negative and autofluorescence controls, the sections were incubated with PBS and normal rabbit or mouse serum instead of the specific antibody. The nuclei were counterstained with 0.4 mM/ml 4’,6-Diamidino-2-Phenylindole, Dihydrochloride (DAPI; Molecular ProbesTM, Invitrogen, Eugene, OR, USA) for 15 min at room temperature. Finally, the specimens were mounted in buffered glycerol and their images were visualized in an immunofluorescence microscopy (OLYMPUS BX51).

### QuPath Immune Matricellular Score

To measure the IHC expression of each different marker and quantify protein expression, the TMA slides were digitally scanned at ×40 magnification using a Panoramic 250 whole slide scanner (3DHistech, Budapest, Hungary). To calculate the immune matricellular score, the stained TMA sections were analyzed using a modified and adapted methodology in software QuPath (version 0.2.1; Centre for Cancer Research & Cell Biology, University of Edinburgh, Edinburgh, Scotland), performed recently by our group to evaluate immune matricellular components in malignant mesothelioma, lung cancer, and breast cancer (21, 22). The modified methodology in QuPath facilitated the measures, was less time-consuming, and reduced intra- and inter-observer differences. In briefly, all cores were evaluated during the scoring process to manually exclude invalid cores (less than 10% of tumor per core or artifact).

TMAs were quantified on QuPath using a simple automated, semi-assisted method. First, each scanned TMA slide was submitted to a series of automated evaluations: staining vector analysis, total tissue area detection, tumor separation from non-tumor areas, and cellular detection. Next, we established the threshold of positivity for each one of our markers through trial and error and the cells considered to be positive were submitted to validation by an expert pathologist before being applied to the full array. Finally, QuPath outputted the number of positive cells per mm^2^ of tissue. We refer to low expression as positive cell density equal to or below the median expression level in the cohort, and high expression as positive cell density above this median.

### Data Management and Statistical Analysis

For the clinical data, either a Pearson chi-square test or a Fisher’s exact test was used to compare categorical variables. Differences in continuous immunohistochemistry variables between the different histological types of the patients were detected using Kruskal-Wallis and post-hoc tests, and the significance values were adjusted by Bonferroni’s correction for multiple tests. Initial analyses of OS were done using Kaplan-Meier curves and compared by log-rank test. Final multivariate analyses were done using the stepwise Cox proportional hazard model including known variables with implications on prognosis, such as histologic types and TNM pathological stage, and all the immune-matricellular variables evaluated. In addition, the general linear model was used to test the relationship between one continuous variable and several others, and the residuals were examined to ensure that they were approximately normally distributed. The statistical software program IBM SPSS (version 22; Armonk, NY, USA) performed the computations for all analyses. P*-*value < 0.05 was considered significant.

## Results

### Clinicopathologic Characteristics of the Study Cohort

**Table 1** summarizes the demographic and clinicopathological features of patients. The median age of patients was 65 years (range, 30–88 years). Sixty-six patients were male (55%), and 54 were female (45%). Most patients were either smokers or former smokers (79.8%), and the median smoking load of these patients was 53.9 packs/year (range, 1.5–150).

**Table 1:** Demographic and clinicopathologic characteristics of the patients (N = 120).

Characteristics	Number (%) of patients
**Gender**	
*Male*	66 (55.0%)
*Female*	54 (45.0%)
**Age (years)**	
*Median (range)*	65 (30–88)
*≤65*	64 (53.3%)
*>65*	56 (46.7%)
**Smoke status** ^**a**^	
*Smoker/former-smoker*	71 (79.8%)
*Non-smoker*	18 (20.2%)
**Smoking load (pack/years)**	
*Mean (range)*	53.9 (1.5–150)
**Histological subtypes**	
*Adenocarcinoma*	73 (60.8%)
*Squamous cell carcinoma*	40 (33.3%)
*Large cell carcinoma*	7 (5.8%)
**Tumor size (cm)**	
*Mean (range)*	4.46 (1–13)
**T stage** ^†^	
*T1*	29 (24.2%)
*T2*	62 (51.7%)
*T3*	23 (19.2%)
*T4*	6 (5.0%)
**N stage** ^†^	
*N0*	80 (66.7%)
*N1*	24 (20.0%)
*N2*	16 (13.3%)
**M stage** ^a^ ^†^	
*M0*	65 (58.0%)
*M1*	47 (42.0%)
**Pathological stage** ^†^	
*I*	47 (39.2%)
*II*	27 (22.5%)
*IIIA*	42 (35.0%)
*IV*	4 (3.3%)
**Metastases site** ^a^	
*Brain*	22 (19.6%)
*Bone*	6 (5.4%)
*Pleura*	6 (5.4%)
*Lung*	5 (4.5%)
*Liver*	3 (2.7%)
*Kidney*	2 (1.8%)
*Breast*	1 (0.9%)
*Spine*	1 (0.9%)
**Recurrence** ^a^	15 (13.3%)
**Adjuvant treatment**	
***Chemotherapy***	
*No*	65 (54.2%)
*Yes*	55 (45.8%)
***Radiotherapy***	
*No*	88 (73.3%)
*Yes*	32 (26.7%)
**Status** ^a^	
*Death*	63 (53.8%)
**Follow up (months)**	
*Mean (range)*	40.7 (0–126)

a Some cases had missing follow-up information: smoke status (31); M stage (8); Recurrence (7); Metastases site (8); Status (3).

^†^8th Edition International Association for the Study of Lung Cancer (18).

The tumors were histologically classified as an adenocarcinoma in 73 patients (60.8%), a squamous cell carcinoma in 40 patients (33.3%), and a large cell carcinoma in 7 patients (5.8%). Pathological examinations of the surgically resected lung tissue confirmed 47 patients in stage I (39.2%), 27 in stage II (22.5%), 42 in stage IIIA (35.0%), and 4 patients who upgraded to stage IV (3.3%).

Early NSCLC disease was detected in the large majority of cases—29 were identified as a T1 stage (24.2%) and 62 as a T2 stage (51.7%). Additionally, we detected N0 stage in 80 patients (66.7%) and M0 in 65 of them (58.0%). Conversely, among the advanced NSCLC cases, 23 were T3 stage (19.2%), and 6 were T4 stage (5.0%), 24 were N1 stage (20.0%), 16 were N2 stage (13.3%), and 47 were M1 stage (42%). In decreasing order of frequency, brain metastases occurred in 22 patients (19.6%), bone and pleura occur in 6 patients, each 1 (5.4%), contralateral lung in 5 (4.5%), liver in 3 (2.7%), kidney in 2 (1.8%), and breast and spine in 1 patient each (0.9%). Disease recurrence occurred in 15 patients (13.3%).

Fifty-five patients (45.8%) received adjuvant chemotherapy, whereas 32 patients (26.7%) received brain radiotherapy. During the follow-up (mean, 40.7 months; range, 0–126 months), 63 patients (53.8%) died due to widespread metastases, evidence that some NSCLCs developed occult spread beyond the lung even after “curative” surgical resection. Our next step was thus to explore which tumors are destined to relapse and metastasize, and whose patients could benefit from adjuvant therapy and perhaps eradicate any residual tumor.

### Qualitative and Quantitative Characterization of Functional Immune Cells

As expected, quantification showed that the most prevalent immune cellular proteins in this NSCLC cohort were T cells CD3+ (median, 278.50 cell/mm^2^; interquartile range (IQR), 504.99 cell/mm^2^), cytotoxic T cells [CD8^+^ (median, 217.38 cell/mm^2^; IQR, 417.23 cell/mm^2^), and Granzyme B (median, 6.19 cell/mm^2^; IQR, 19.50 cell/mm^2^)]. We also observed the presence of immune checkpoints PD-L1 (median, 0.37 cell/mm^2^; IQR, 2.84 cell/mm^2^), LAG-3 (median, 95.33 cell/mm^2^; IQR, 308.70 cell/mm^2^), CTLA-4+ (median, 227.50 cell/mm^2^; IQR, 186.39 cell/mm^2^), and VISTA 1 (median, 52.86 cell/mm^2^; IQR, 144.85 cell/mm^2^), as well as macrophages CD68+ (median, 225.78 cell/mm^2^; IQR, 107.62 cell/mm^2^), regulatory T cells CD4+ (median, 98.32 cell/mm^2^; IQR, 310.50 cell/mm^2^), FOXP3+ (median 127.00 cell/mm^2^; IQR, 109.47 cell/mm^2^), NK cells CD57+ (median 116.55 cell/mm^2^; IQR, 149.42 cell/mm^2^), and B lymphocytes CD20+ (median, 154.18 cell/mm^2^; IQR, 359.69 cell/mm^2^) (**Supplementary Table S1**).

**Figure 2:**
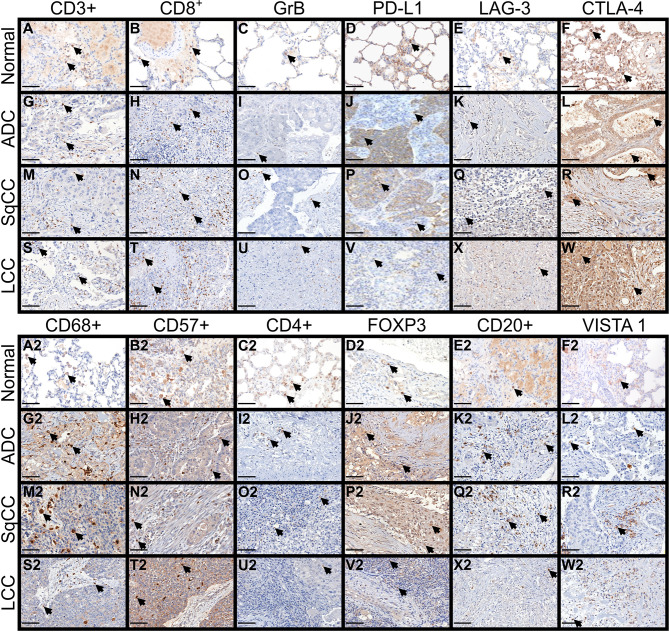
Microphotographs of representative examples of the 12 immune markers in lung ADC, SqCC, and LCC specimens, divided into three compartments: normal tissue **(A–F, A2–F2)**, tumor tissue, and intermediate tissue **(G–W, G2–W2)**. Arrows indicate cells expressing each immune marker (brown). **(A–F, A2–F2)** In control tissue, focal TLIs along of the alveolar septa are visible expressing the immune markers. **(G–W)** In tumor tissue, note the moderately expression of CD3+, CD8^+^, PD-L1, LAG-3 and CTLA-4+ by tumor and stromal TILs of ADC, SqCC, and LCC. **(G2–W2)** inferior panel showing a strong expression of CD68+, CD57+ and FOXP3+, and a moderate expression of CD20+ and VISTA 1. **(C, I, O, U)** No notable staining for Granzyme B was observed in TILs of the control and tumor tissue. ADC, adenocarcinoma; LCC, large cell carcinoma; SqCC, squamous cell carcinoma; GrB, Granzyme B; PD-L1, programmed death ligand 1; LAG-3, lymphocyte activating gene 3; CTLA-4+, Cytotoxic T-Lymphocyte Associated Protein 4; FOXP3+, Fork head box protein P3; VISTA 1, V-domain Ig suppressor of T cell activation. Scale bars: X400, 50 µm.

**Figure 2** shows the immune cell mapping according to histologic subtypes, in immune stained sections containing normal tissue (A to F, and A2 to F2), and tumor tissue and tumor intermediate portion (G to W, and G2 to W2). Overall, the amount of cytotoxic T cells CD8^+^, TILs CTLA-4+, and macrophages CD68+ was prominent in the tumor tissue than in the normal tissue and intermediated portion of the tumor, as were the amount of LAG-3, CTLA-4+, macrophages CD68+, and NK cells CD57+, while malignant PD-L1 cells were moderately distributed. Observing the **Figure 2**, according to histotypes, T cells CD3+ and cytotoxic T cells (CD8^+^, Granzyme B) were more prominent in LCC compared to adenocarcinoma and squamous cell carcinoma, whereas macrophages CD68+ and VISTA1 predominated in adenocarcinoma and squamous cell carcinoma compared to large cell carcinoma.

**Figure 3:**
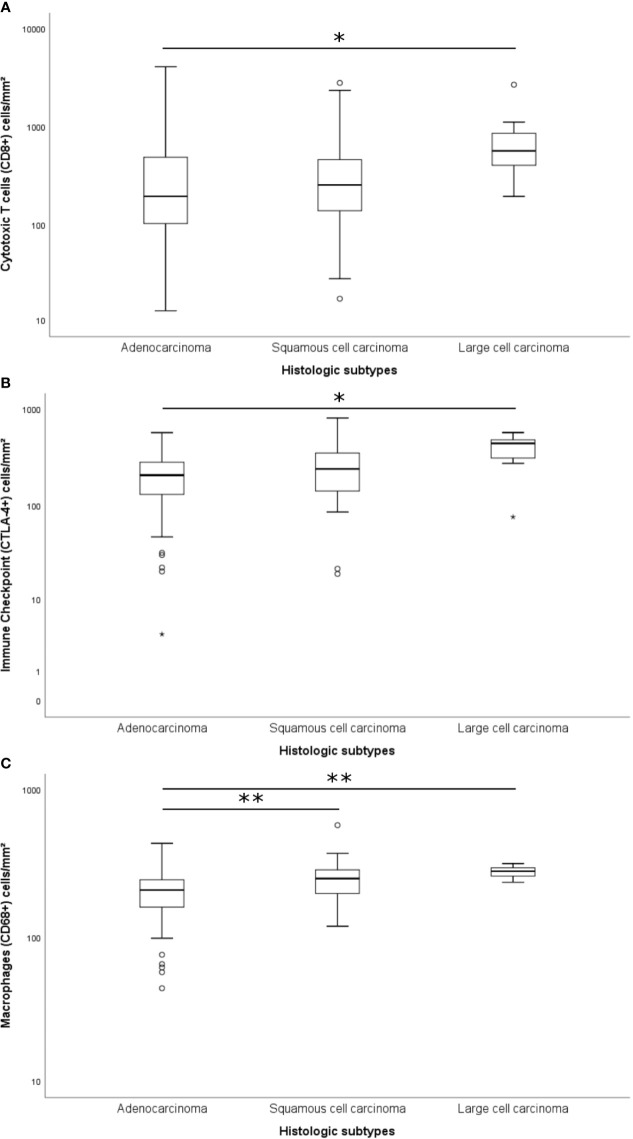
Box plots of differentially expressed immune proteins among histological subtypes. Only the cytotoxic T cells CD8^+^ (**A**; P = 0.034; Adjusted P-value [ADC-LCC] = 0.034), the immune-checkpoint CTLA-4+ (**B**; P = 0.012; Adjusted P-value [ADC-LCC] = 0.024), and the macrophages CD68+ (**C**; P < 0.001; Adjusted P-value [ADC-SqCC] = 0.005, and Adjusted P-value [ADC-LCC] = 0.007) presented significative difference between NSCLC subtypes. The y-axis was shown on the log scale. The association between immune cells and histological types was calculated by the non-parametric Kruskal-Wallis test. The horizontal bars represent the statistical difference between groups; the pairwise comparison was adjusted by Bonferroni’s correction for multiple tests (Adjusted P-value). (*) was used when P > 0.05, and (**) was used when P ≥ 0.01. ADC, adenocarcinoma; LCC, large cell carcinoma; SqCC, squamous cell carcinoma; CTLA-4, Cytotoxic T-Lymphocyte Associated Protein 4.

The immune cell mapping coincided with the quantification of TILs (**Supplementary Table S2**; Kruskal-Wallis’ test). A significant difference was found between histologic subtypes and cytotoxic T cells CD8^+^ (P = 0.034), TILs CTLA-4+ (P = 0.012), and macrophages CD68+ (P < 0.001) (**Figure 3**). We observed a statistical difference in the distribution of cytotoxic T cells CD8^+^ and TILs CTLA-4+ between adenocarcinomas and large cell carcinomas (P = 0.034 and P = 0.024, respectively), whereas the distribution of macrophages CD68+ differed both between adenocarcinomas and large cell carcinomas (P = 0.007) and adenocarcinomas and squamous cell carcinoma (P = 0.005).

We then investigated whether distinct TILs barriers could be identified in the tumor and intermediate portions. Our analysis showed that the further from malignant cells, the number of TILs phenotypes was higher. We also identified two distinct functional immune-cellular barriers—the first, involving tumor portion, showed proximity between malignant cells and T cells, and the second, relating to intermediate portion of the tumor, showed distant proximity between non-cohesive nests of malignant cells and T cells. When we assessed the distribution pattern of specific key TILs phenotypes, the first immune-cellular barrier coincided with a higher presence of CD3+, CD8^+^, PD-L1, and CTLA-4 (**Figures 2G, M, S; H, N, T, J, P, V, L, R, W**), whereas the second barrier involved the presence of regulatory T cells (CD4+ and FOXP3+) at a lower frequency than that of their cytotoxic T-cell counterparts (**Figures 2I2, O2, U2; J2, P2, V2**). We observed that these two barriers of immune cellular distribution were frequently repeated and that regulatory T cells work as a fragile barrier compared to that of their cytotoxic T-cell counterparts.

### Associations Between Functional Immune Cells

**Figure 4:**
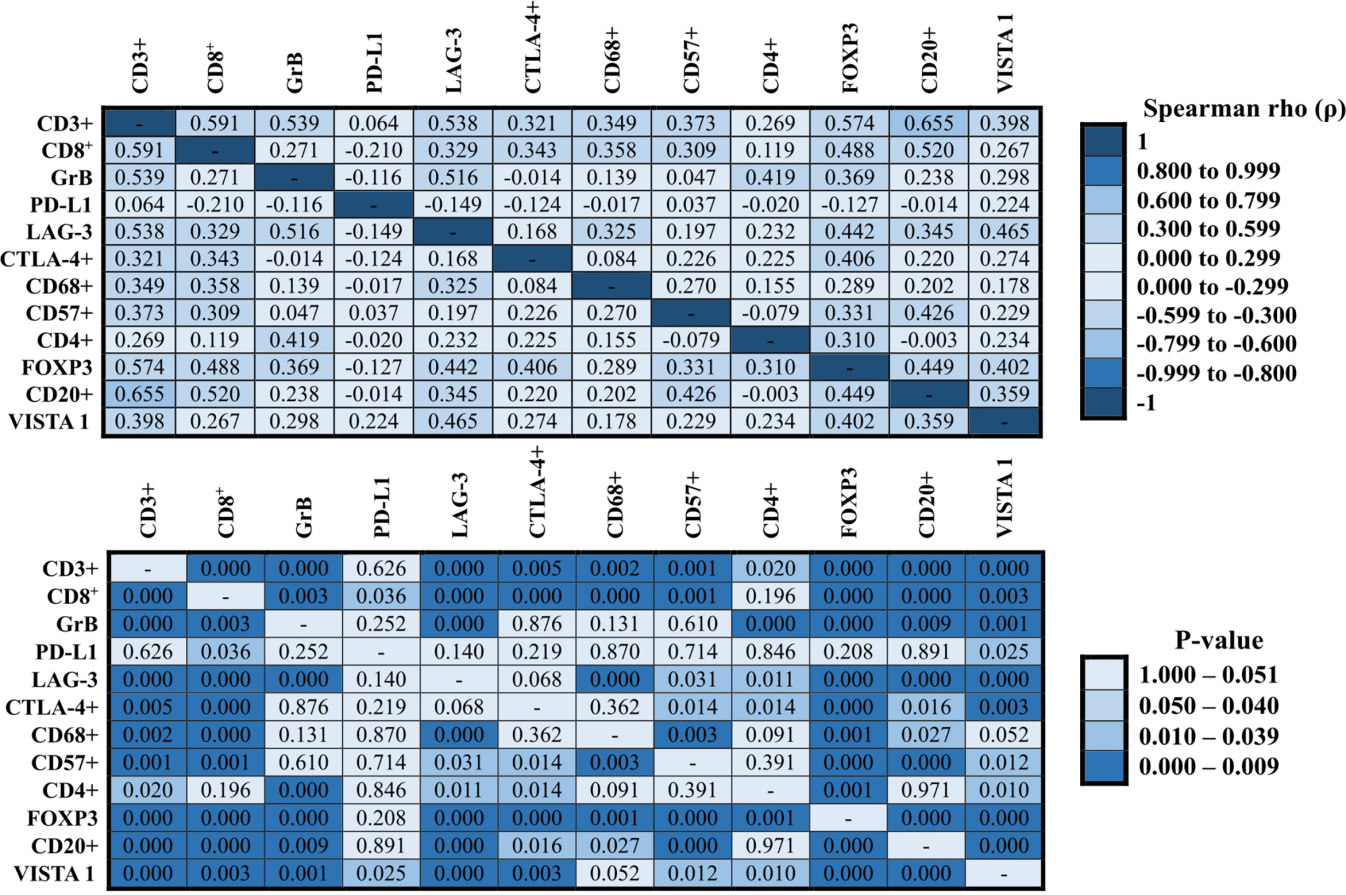
Correlation between immune proteins expressed in NCLSC. The heat-maps presented the correlation values. The superior heat-map presents the results for Spearman’s rho, and the bottom heat-map presents the equivalent P-values (Spearman’s test).

A heatmap was constructed to illustrate the correlations between the expressions of the immune cells (**Figure 4**, Spearman’ test). The best correlation (strong correlation, ρ ≥ 0.6) was observed between T cells CD3+ and B lymphocytes CD20+ (ρ = 0.655, P = 0.000). However, we also observed other important correlations (0.5 ≥ ρ > 0.59), as follows: T cells CD3+ and cytotoxic T cells CD8^+^ (ρ = 0.591, P = 0.000), T cells CD3+ and cytotoxic T cells Granzyme B (ρ = 0.539, P = 0.000), T cells CD3+ and TILs LAG-3 (ρ = 0.538, P = 0.000), T cells CD3+ and regulatory T cells FOXP3 (ρ = 0.574, P = 0.000), cytotoxic T cells CD8^+^ and B lymphocytes CD20+ (ρ = 0.520, P = 0.000), and cytotoxic T cells Granzyme B and TILs LAG-3 (ρ = 0.516, P = 0.000). Many other correlations were considered as moderate correlations (**Figure 4**, 0.3 ≥ ρ > 0.59). In contrast, except for the weak negative correlation between cytotoxic T cells CD8^+^ and malignant cells PD-L1 that presented statistical significance (ρ = -0.210, P = 0.036), none other negative correlation was statistically significant.

### Qualitative and Quantitative Characterization of Matricellular Mechanical Proteins

For collagen types, the quantification showed that Col III was the dominant protein associated with matricellular barrier (median, 24.89 fibers/mm^2^; IQR, 11.15 fibers/mm^2^), followed by Col V (median, 13.18 fibers/mm^2^; IQR, 8.20 fibers/mm^2^), Col I (median, 2.54 fibers/mm^2^; IQR, 2.71 fibers/mm^2^), and CAFs actin+/vimentin+ (median, 12.39 fibers/mm^2^; IQR, 7.23 fibers/mm^2^). As expected, these four protein types prevailed in the TME and were equally distributed between adenocarcinomas, squamous cell carcinomas, and large cell carcinomas (**Supplementary Table S1**).

**Figure 5:**
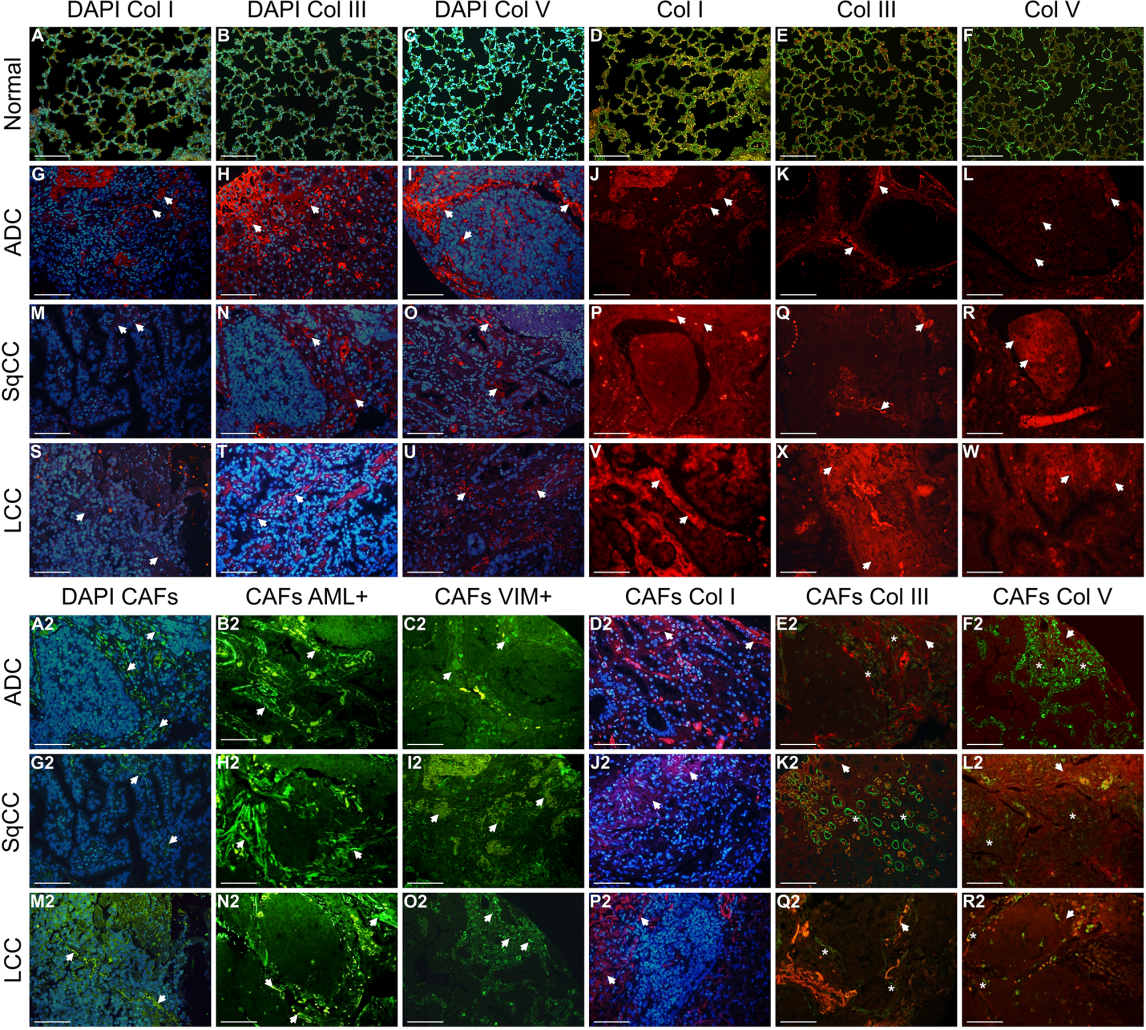
Immunofluorescent staining of representative examples of the four matricellular markers in lung ADC, SqCC, and LCC specimens, divided into three compartments: normal tissue **(A–F)**, tumor tissue, and intermediate tissue **(G–W, A2–R2)**. Arrows indicate cell and fibers expressing each matricellular marker (blue, DAPI; Col fibers, red; CAFs, green; merge CAFs and Col). **(A–F)** In normal lung tissue, green birefringence of Col types are visible expressing along of the alveolar septa. **(G–W)** In tumor tissue, note a similar red birefringence of Col types in the stroma of the three histotypes. **(A2–R2)** Intermediate portion of the tumor showing a strong green birefringence of CAFs and red birefringence of Col types. Note the colocalization of CAFs and Col types in the stroma of the intermediate portion of the tumor. Arrows: Col fibers; asterisks: CAFs. ADC, adenocarcinoma; LCC, large cell carcinoma; SqCC, squamous cell carcinoma; Col I, collagen type I; Col III, collagen type III; Col V, collagen type V; CAFs, cancer-associated fibroblasts. Scale bars: X400, 20 µm.

**Figure 5** shows **t**he matricellular mapping according to histologic subtypes, in immune stained sections visualized under fluorescence, and containing normal tissue (A to F), and tumor tissue and tumor intermediate portion (G to W, and A2 to R2, respectively). Overall, the amount of Col I, Col III, Col V, and CAFs was similar in normal portion, tumor, and intermediate portion of the three histotypes. However, the Col I, III, V, and CAF phenotypes were different among adenocarcinomas, squamous cell carcinomas, and large cell carcinomas. Under immunofluorescence, adenocarcinomas exhibited a focal red birefringence of Col I (**Figure 5J**) and Col V (**Figure 5L**) and a diffuse strong red birefringence of Col III fibers (**Figure 5E**). Their collagen fibers were organized in a dense but irregular fibrillary pattern—while Col I and Col III fibers surrounded groups of malignant glands, Col V fibers enveloped malignant cells individually. In squamous cell carcinomas, the immunofluorescence showed a weak red birefringence of Col I (**Figure 5P**), III (**Figure 5Q**), and V (**Figure 5R**) fibers, which either surrounded layers of squamous malignant cells in a focal, irregular fibrillar pattern (Col I and III) or were organized around individual malignant cells (Col V). Finally, in large cell carcinomas, we observed an equally dense and strong red birefringence of Col I (**Figure 5V**), III (**Figure 5X**), and V (**Figure 5W**) fibers under immunofluorescence, forming an irregular texture of thin fibers and individual large groups of malignant cells. Conversely, CAFs actin+/vimentin+ exhibited a green birefringence in the immunofluorescence and assumed a very similar distribution to that of Col I, III, and V in all three histological NSCLC types.

We also identified three tumor-associated collagen barriers (TACB) corresponding to different levels of collagen fibers’ reorganization to locate and characterize malignant cells. In the first TACB, we observed a localized increase in CAFs and a low deposition of Col without obvious orientation near the malignant cells. This barrier was also characterized by a homogeneous distribution but low proximity between the malignant cells and the Col fibers and CAFs. Next, in the second TACB, the collagen fibers were aligned in parallel to the tumor border, thus suggesting a moderate barrier between the malignant cells and the Col fibers and CAFs. Finally, in the third TACB, a high amount of Col fibers and CAFs were bundled and aligned perpendicularly to the tumor border, a sign of prominent barrier between these proteins and malignant cells.

When we assessed the distribution pattern of specific Col and CAF phenotypes, the Col I fibers coincided with the second TACB, while Col III coincided with the third TACB, which suggests that these Col types might be stronger malignant cells barrier (**Figures 5D2, J2, P2**). Col V fibers and CAFs, on the other hand, were found at a lower frequency than their Col I and Col III counterparts and coincided with the first TACB, thus suggesting that these fibrillar proteins work as fragile malignant cells barrier (**Figures 5L2, R2**). As with the TILs barriers, here also the three signatures of matricellular distribution were recurrent and showed that Col V and matched CAFs fibers work as an inverse barrier to that of their Col I and Col III equivalents, impacting their level of proximity with malignant cells in opposite ways. Overall, this suggested that Col I and III/CAFs works as effective barrier against malignant cells, thus hindering invasion of TME and metastases, whereas Col V/CAFs exhibited an unproductive barrier against malignant cells, thus favoring TME invasion and metastases.

### Association Between Mechanical Matricellular Proteins and Functional Immune Cells

**Table 2** shows the correlation between collagen types and matched CAFs with immune cells. A negative correlation was found between Col I and TILs VISTA 1 (ρ = -0.188; P = 0.040), as well as Col V and NK cells CD57+ (ρ = -0.193; P = 0.035). In contrast, a positive correlation was found between Col III and T cells CD3+ (ρ = 0.367; P = 0.001), Col III and TILs LAG-3 (ρ = 0.218; P = 0.017), Col III and cytotoxic T cell Granzyme B (ρ = 0.270; P = 0.003), regulatory T cells CD4+ (ρ = 0.215; P = 0.018), and macrophages CD68+ (ρ = 0.205; P = 0.025). CAFs were positively correlated with cytotoxic T cell Granzyme B (ρ = 0.219; P = 0.016), Col III (ρ = 0.278; P = 0.002), and Col V (ρ = 0.475; P = 0.000). We also observed a positive correlation between Col III and Col V (ρ = 0.263; P = 0.004). These findings can suggest that Col I, Col III, and CAFs are an effective barrier against malignant cells and prevent invasion of TME and metastases, whereas Col V is a useless barrier against malignant cells and favors TME invasion and metastases.

**Table 2:** Correlation between immune cells, collagen types, and cancer-associated fibroblasts (N = 120; Spearman correlate, P < 0.05).

	Col I		Col III		Col V		CAFs	
	ρ	P-value	ρ	P-value	ρ	P-value	ρ	P-value
T cells CD3+^a^	-0.004	0.975	0.367	**0.001**	0.060	0.610	0.051	0.667
Cytotoxic T cells CD8^+a^	0.067	0.470	-0.065	0.481	-0.065	0.479	0.033	0.720
Cytotoxic T cells Granzyme B	-0.098	0.289	0.270	**0.003**	0.058	0.531	0.219	**0.016**
Malignant cells PD-L1	0.108	0.285	0.122	0.227	0.179	0.074	0.076	0.451
TILs LAG-3	-0.087	0.343	0.218	**0.017**	0.011	0.904	0.130	0.157
TILs CTLA-4+^a^	-0.045	0.624	-0.121	0.192	0.028	0.766	-0.046	0.616
Macrophages CD68+	0.156	0.089	0.205	**0.025**	-0.058	0.530	0.061	0.508
Natural killer cells CD57+	0.013	0.891	0.082	0.374	-0.193	**0.035**	-0.039	0.673
Regulatory T cells CD4+	0.045	0.629	0.215	**0.018**	0.057	0.534	0.144	0.116
Regulatory T cells FOXP3	-0.046	0.621	0.099	0.284	0.089	0.336	0.032	0.725
B Lymphocytes CD20+	-0.029	0.755	-0.039	0.675	-0.102	0.269	-0.068	0.462
TILs VISTA 1	-0.188	**0.040**	0.090	0.328	0.003	0.975	-0.002	0.981
Col I	–	**-**	0.090	0.330	-0.078	0.397	-0.035	0.708
Col III	0.090	0.330	–	–	0.263	**0.004**	0.278	**0.002**
Col V	-0.078	0.397	0.263	**0.004**	–	–	0.475	**0.000**
CAFs	-0.035	0.708	0.278	**0.002**	0.475	**0.000**	–	–

a Missing information: CD3+ (46); CD8^+^ (1); PD-L1 (20); CTLA-4+ (1).

TILs, tumor infiltrating lymphocytes; PD-L1, programmed death ligand 1; LAG-3, lymphocyte activating gene 3; CTLA-4+, Cytotoxic T-Lymphocyte Associated Protein 4; FOXP3, Fork head box protein P3; VISTA 1, V-domain Ig suppressor of T cell activation; Col I, collagen type I; Col III, collagen type III; Col V, collagen type V; CAFs, cancer-associated fibroblasts.

Bolded values refer to a P-value with statistical significance (P < 0.05).

### Association Between Immune Matricellular Barriers and TNM Stage

**Figure 6:**
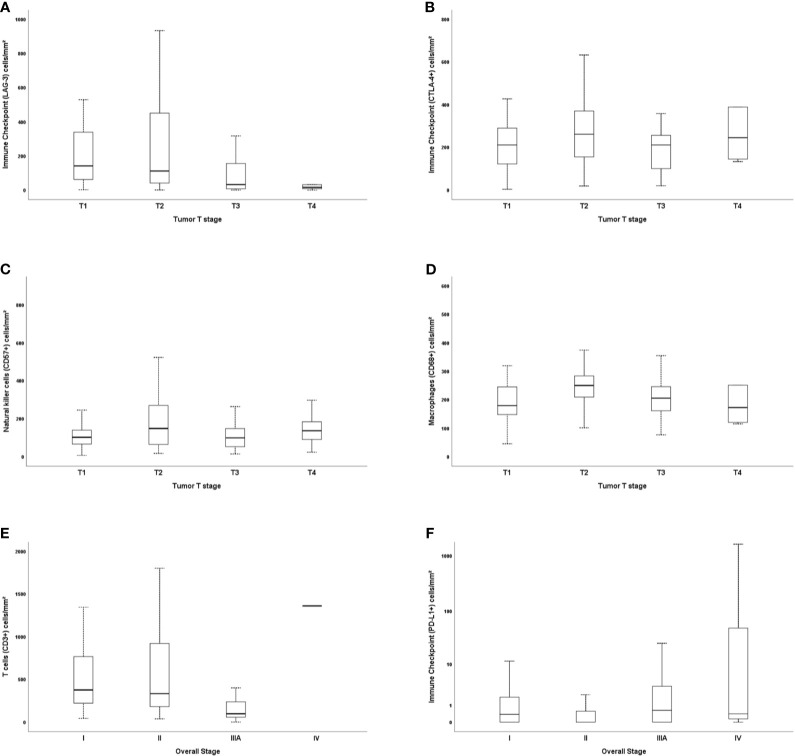
Box plots demonstrating the relationships between immunostaining for immune cells, tumor T stage, and overall stage. LAG-3 and T stage **(A)**, CTLA-4+ and T stage **(B)**, natural killer cells CD57+ and T stage **(C)**, macrophages CD68+ and T stage **(D)**, and CD3+ and overall stage **(E)**, as well as PD-L1 **(F)**. The solid bars represent the number of immune cells between the 25th and 75th percentiles; the black bar shows the median value; and the top and bottom brackets show the extreme values.

The level of staining for immune matricellular-associated barriers was significantly associated with factors related to the stage of the tumor. The general linear model analysis demonstrated that staining for T cells (CD3+) and malignant cells PD-L1 were associated with the overall stage (P = 0.019), whereas TILs LAG-3 and CTLA-4+, NK cells (CD57+), and macrophages (CD68+) were associated with the presence of T2 tumor stage (P = 0.04, P = 0.01, P = 0.01, and P = 0.003; respectively). The statistical significance of these relationships was seen not only in their individual univariate analyses, but also under a multivariable model. However, we found no association between N stage and these proteins. **Figure 6** uses six plots to demonstrate the relationships between the staining for immune matricellular-associated proteins and T stage (**Figures 6A–D**), or overall stage (**Figures 6E, F**). The six box plots show a relatively weak relationship between protein expression and stage factors.

### Survival Analysis

**Figure 7:**
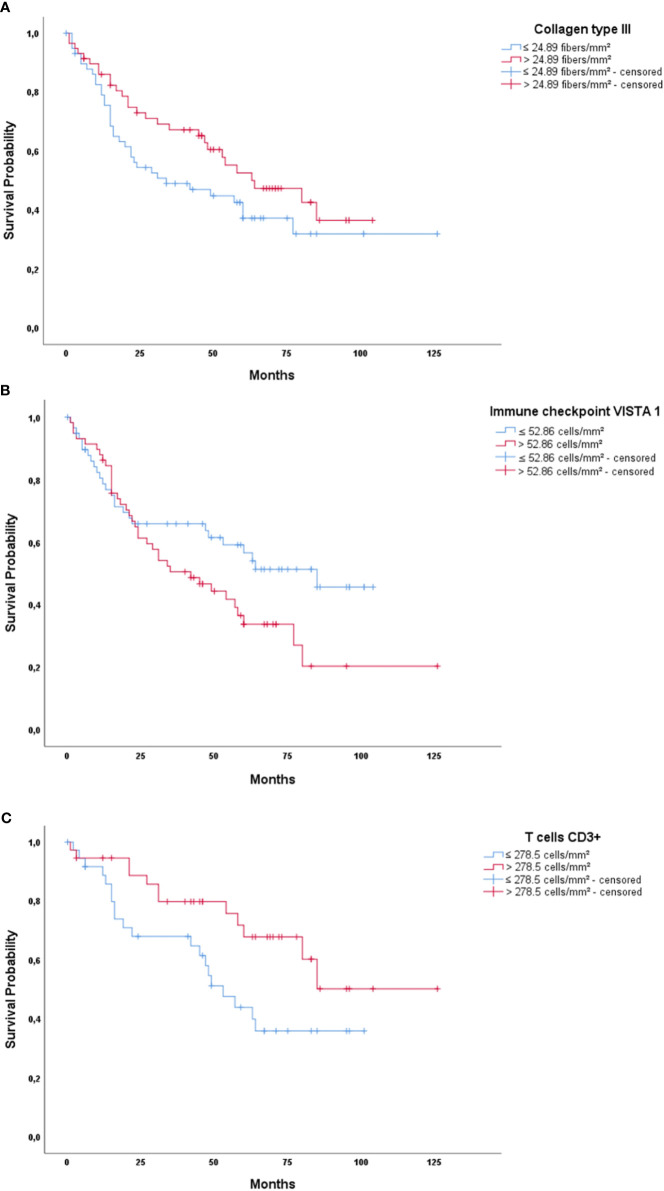
Kaplan-Meier plots of survival probability versus follow-up time in months in those patients with pathological early **(A)** and advanced stage disease **(B, C)**. **(A)** Col III in early disease patients showing better survival for patients with high Col III expression. **(B)** Immune checkpoint VISTA 1 in advanced stage patients, showing better survival for patients with low VISTA 1 expression. **(C)** T cells CD3+ in advanced stage patients, showing better survival time for patients with high CD3+ expression.

We then evaluated the impact of these proteins on survival, controlled for TNM stage. Before elaborating the multivariate model, we tested the variables using Cox’s univariate model (see **Supplementary Table S3**). Next, in the multivariate model, our first examination was performed in patients who had pathological stage I, II, and III tumors. We conducted a stepwise multivariate Cox regression analysis where we inputted variables with known impact on prognosis, such as histologic types and TNM pathological stage, and then added all the immune-matricellular variables that were being assessed. **Table 3A** brings the results of the multivariate model analysis. Just two variables were significantly associated with survival time—pathological stage and staining of the tumor for Col III—which were then used as a categorical variable. Once these two variables were accounted for, none of the others showed any association to survival. For instance, although we found that individual T and N stages were significantly related to survival time in the absence of Col III (P ranging from 0.017 to 0.028), when Col III was present as a covariate, this relationship became much stronger. Given that the median density of Col III fibers in our cohort was 24.89 fibers/mm^2^, we found a tendency that separated patients into two groups with distinctly different average survival times as illustrated by the top Kaplan-Meier plot in **Figure 7A**. The top curve represents patients expressing >24.89 Col III fibers/mm², whose median survival time after surgery was quite long (64.7 months). By contrast, those with ≤24.89 fibers/mm², seen in the bottom curve, had a median survival time of 59.6 months after surgery (P = 0.08 by Breslow test).

**Table 3A:** Cox proportional hazard model analysis of survival time, controlled for tumor pathological stages I, II, and III.

Variable	Coefficient	SE	P-value
**Stage**			
*I*	-1.929	0.732	**0.008**
*II*	0.081	0.599	**0.002**
*III (reference)*			
**Collagen type III**			
*>24.89 fibers/mm^2^*	-0.120	0.037	**0.001**
*≤24.89 fibers/mm^2^ (reference)*			

SE stands for the standard error of the coefficient.

Bolded values refer to a P-value with statistical significance (P < 0.05).

Subsequently, we examined the importance of immune matricellular-associated barrier to survival only in patients who had progressed to pathological stage IV (**Table 3B**). In this scenario, after controlling for the pathological stage, it was T cell (CD3+) and immune checkpoint (VISTA 1) densities which significantly related to survival (P = 0.004 and P = 0.016, respectively). Their median values were 278.5 cells/mm^2^ for CD3+ and 52.86 cells/mm^2^ for VISTA 1, and although there is no biologically distinctive cut point in CD3+ and VISTA 1, we chose these median values as a practical way to examine the outcome of stage IV patients. We found a tendency that these mean values separated these patients into two groups with distinctly different average survival times, also seen in **Figure 7**. The group with ≤ 52.86 cells/mm² VISTA 1 appears as the top curve in the second Kaplan-Meier plot, and their median survival time was 64 months. By contrast, those with >52.86 cells/mm² VISTA 1 (bottom curve) had a median survival time of 53 months (P = 0.06 by log-rank test). CD3+ values are seen in the bottom plot, whose top curve represents the group with CD3+ > 278.5 cells/mm² and their median survival of 88.7 months, whereas the group with CD3+ values ≤ 278.5 cells/mm² appears as the bottom curve and their median survival was just 57.3 months (P = 0.036 by log-rank test).

**Table 3B:** Cox proportional hazard model analysis of survival time, controlled for tumor pathological stage IV.

Variable	Coefficient	SE	P-value
**Stage**			
*I*	-2.658	1.132	**0.019**
*II*	-0.094	1.095	0.409
*III*	-1.746	1.157	0.131
*IV (reference)*			**0.003**
**T cells (CD3+)**			
*>278.5 cells/mm^2^*	-1.391	0.491	**0.005**
*≤278.5 cells/mm^2^ (reference)*			
**Immune checkpoint (VISTA 1)**			
*≤52.86 cells/mm^2^*	1.057	0.438	**0.016**
*>52.86 cells/mm^2^ (reference)*			

SE stands for the standard error of the coefficient.

Bolded values refer to a P-value with statistical significance (P < 0.05).

## Discussion

Clearly, examining the clinicopathologic characteristics of our study cohort of patients, we noticed that the probable reason that surgery resection fails to cure some patients with early-stage NSCLC is because of micrometastases that have not yet been detected by either routine imaging or routine pathological analysis. In fact, examinations of the surgically resected lung tissue confirmed 96% of the patients in the early stage. Among these, 34% developed systemic metastases during follow-up and, 13% presented disease recurrence. Therefore, the question of interest is whether additional, more technological and biological information gathered from either the tumor tissue or its milieu to be integrated with the classic TNM stage classification can help us to improve risk stratification and patient selection for adjuvant systemic treatment.

The development of cancer cell invasion and metastases certainly encompasses a series of complex, sequential stages, but among these, the TME is thought to be important because tumor-reprogrammed lung microenvironment promotes both primary lung tumors and metastases by contributing mainly with mechanical and functional barriers (5). In particular, immune cell infiltration can be restricted by multiple barriers in immune privilege landscape. These may comprise physical or mechanical barriers created by TME combined with different initiating cell types, including cancer-associated fibroblasts (CAFs) and matricellular collagen types, or functional barriers created by immunosuppressive molecules including PD-L1, LAG-3, CTLA-4, and VISTA 1 (23). Therefore, in order to understand the relationship between immune-matricellular barriers and metastatic process, we used a two-stage design. First, we used IHC and IF respectively to characterize immune phenotypes, CAFs, and fibrillar collagen types in TME of adenocarcinomas, squamous cell carcinomas, and large cell carcinomas histological subtypes. Second, we examined the clinical association between the immune-matricellular barriers in TMA from 120 patients with surgically excised NSCLC. We provide new evidence that NSCLC cells can express matricellular proteins with known mechanical barrier function, and that expression of those proteins demand prominent immune signatures and with significantly longer overall patient survival.

While most of the studies evaluated immune cells only in the tumor tissue portion, we described the tumor immune microenvironment as a whole at the normal tissue, tumor intermediate portion, and tumor tissue. Another main innovative feature of our study was the description of the relationship between immune-matricellular barriers with TNM stage and risk of death. Overall, we showed that immunosuppressive factors present in the intermediate portion of TME such as regulatory T cells created a functional barrier against T cells infiltration, enhanced by lack of activated T cells CD8, and demand of immunosuppressive checkpoints in tumor portion barrier, thus facilitating tumor progression. We also found that the functional barrier between immune cells and malignant cells was also reinforced by tumor-associated collagen mechanical barrier corresponding to different levels of collagen fibers reorganization to locate and characterize malignant cells. In this scenario, we inferred that Col I and III/CAFs also have a high chance to create a mechanical barrier against malignant cells and prevent the invasion of the TME, whereas Col V/CAFs has a low chance of creating a mechanical barrier favoring TME invasion. In fact, while high density of collagen type III was associated with tumors in early stage and better survival rates, tumors expressing high levels of VISTA 1 and low levels of CD3+ identified patients who had progressed to pathological stage IV and presented a poor survival. However, there are some major points, which need to be addressed, as discussed below.

The first important question to discuss is about the significance of the presence of immune cell infiltration for the interaction between collagen types and malignant cells. In short, it is well recognized that macrophages infiltrate the TME in response to tumor-secreted chemotactic signals and exhibit a tumor-supportive phenotype similar to the M2 phenotype (25). These activated macrophages (termed M2) are stimulated primarily by the Th2 cytokines IL-4 and IL-13 and facilitate ECM remodeling, blood vessel formation, and dampen immune activation by secreting cytokines such as IL-10 and TGF-β (25). On the other hand, the collagen distribution and CAFs in the TME contribute to mechano-immunomodulation, which can influence immune cells activation, including macrophages (26). In this way, matrix stiffness activates integrin signaling on macrophages in the TME, resulting in integrin clustering and focal adhesion signaling (27, 28). Collagen types in malignant tissue joint their action with various groups and degrees of myeloid-lineage immune cells, including mast cells, macrophages, and neutrophils, and lymphocytes, including T cells and B cells, to compose mechanical and functional TME barriers with differences in cancer progression (29). For instance, TME barriers in pancreas cancer were categorized as inert, dormant, fibrogenic, or fibrotic based on fibroblasts α-smooth muscle actin (CAFs), Col I and Col III expression; these divergent TME alignments individually influence the amount of CD4+T cells, CD8^+^T cells, macrophages, and neutrophils (30). Increased infiltration of macrophages CD68+ and CD3+ was also described in subcutaneous adipose collagen in gastrointestinal cancer patients with cachexia (31). In breast cancer, increased Col Xα1 in TME joint its action with the low number of total TILs in ER-positive/EGFR2-positive breast cancer (24).

In the present work, we identified three tumor-associated matricellular barriers: the first, with a localized increase in CAFs and a low deposition of Col V without obvious orientation near the malignant cells, the second with increased CAFs and Col I fibers aligned in parallel to the tumor border, and the third with increased CAFs and Col III bundled and aligned perpendicularly to the tumor border, suggesting that these Col types might be stronger malignant cells barrier compared to the fragile Col V counterparts. These results contribute to previous works that have demonstrated that the alignment of the fibrillar components of the ECM has a strong influence on the direction and speed of migrating cells (32). In experimental models, malignant cell invasion was shown to be more precise through engineered *in vitro* frames of linear Col I and Col III than through disorganized frames (33). *In vivo*, local tumor cell invasion was oriented along radially aligned TACS which have been described to locate and characterize tumors (34). When we assessed the distribution pattern of specific key TILs phenotypes, the first immune-cellular barrier coincided tumor portion exhibiting a higher presence of CD3+, PD-L1, LAG-3, CTLA-4, VISTA-1, macrophages, and NK cells, whereas the second immune-cellular barrier involved tumor intermediate portion with the presence of regulatory T cells (CD4+ and FOXP3+). Despite being loyal managers of chemical signals, immune cells and their effectors also employ the molecular sensors of stiffness in response to malignant cells. Recruitment of immune cells and adherent CAFs and directional migration from softer to stiffer regions of the matricellular has been proposed to be a key factor of cancer dissemination (35, 36).

Remarkably, it is important to highlight that we found a low number of CD8 Granzyme B cells compared to CD8^+^ cells in the tumor portion. This observation raises the possibility that in our cohort, most CD8 cells were naïve or inactivated by LAG-3 or TIM3 and promoted a less suppressive immune barrier. We inferred that, on T cells, LAG-3 reduced cytokine and Granzyme production and proliferation while encouraging differentiation into T regulatory cells as previously reported (37, 38). This finding is relevant as recently demonstrated by Zhou and colleagues (39) as far as LAG-3 upregulation after EGFR-TKI failure in advanced NSCLC, suggesting the rational use of LAG-3 inhibitors in advanced NSCLC patients with EGFR mutation. Although cytotoxic T cells were the subpopulation of TILs observed closest to the tumor cells in our NSCLC cohort; other TILs, such as CD4+, macrophages, NK cells, and B lymphocytes, were also found in proximity to the malignant cells. Although we have not investigated dendritic cells in the present work, it is worth remembering that different types of dendritic cells are important to mount an immune reaction (antigen presentation)—if this fails or is inhibited by some types of dendritic cells, the presence of other lymphocytes has no meaning as an effective barrier—excluding NK cells (40–43).

We also found a discrepancy between CD4+ cells and FOXP3+ cells. We inferred that this discrepancy is related to other cells, which theoretically also express FOXP3, such as CD8^+^ T-cell subsets, commonly termed CD8^+^ regulatory T cells, which have been demonstrated to express FOXP3 (28). In addition, some data indicate that FOXP3 could be expressed by some non-lymphoid cells, in particular epithelial cells (27). This was reported to be the case in breast and prostate cancer in particular, where FOXP3 expression was found in normal epithelial cells and its downregulation was related to cancer development. In contrast, FOXP3 was found highly expressed in tumor cells than in corresponding epithelial cells, being proposed its involvement in the biology of cancer. Furthermore, FOXP3 protein expression was detected by immunohistochemistry and western blot in human parenchymal cells from cervical esophageal cancer (27, 44), gastric tumor cells (27, 45, 46), and invasive ductal breast carcinoma (27, 47). It has already been shown that patients with low tumor cells FOXP3 expression and high regulatory T cells count have a significantly worse OS, suggesting that tumor FOXP3 expression could be used as a high-fidelity prognostic potential in NSCLC (28). Further studies using double immunostaining assays are needed to clarify which cell types, other than regulatory T cells, may be expressing the FOXP3 transcription factor in our cohort of NSCLC.

Taken together, our results suggested that the alignment and amount of collagen fibers with T cells emphasize a bimodal cancer progression *via* distinct signaling barriers. In agreement with previous studies (48, 49), our findings suggest that a high-density of Col I and Col III matricellular, rather than a low-density matricellular, decreases immunosuppressive checkpoints and regulatory T cells. Spatially aligned collagen fibers and density around epithelial malignant cells limited the migration of T cells and limited their entry into the tumor mass (50). Other authors did not find an association between Col I and T cell deficiency in pancreatic cancer (51). Of note, in our study, a negative correlation was found between cytotoxic T cells CD8^+^ and malignant cells PD-L1, and previous reports showed that the metastatic urothelial cancer response to anti-PD-L1 treatment involved the movement of CD8^+^ T cells from the cancer portion to the collagen-rich intermediate tumor portion (52). Hitherto, our description of the proximity between malignant cells and LAG-3, activated CD8, regulatory FOXP3, and macrophages with Col III suggest that members of the TGF-β pathway may provide anti-NSCLC-immunity as previously reported by Budhu and colleagues (53).

Another important question arises about the relationship between matricellular collagen and PD-1 on NSCLC progression. Although we did not include PD-1 evaluation in our study design, clinical studies have shown that tumors presenting abundance in T cells are more vulnerable to be controlled by PD-1 blockade. In contrast, tumors in which T cells are present within tumors but not in contact with malignant cells, are refractory to PD-1 blockade (52, 54). In particular, the fibrotic barrier of desmoplastic tumors can cause immuno-suppression through multiple mechanisms (55). In addition, CAFs can have both direct and indirect effects on T cell infiltration and function (55). Recently, a major role for the TGFβ signaling pathway in promoting T cell exclusion from tumor cells has been demonstrated. In breast and colorectal mouse tumor models, neutralizing antibodies against TGFβ were shown to reduce Col I production, incapacitating the T cell excluded profile and increasing the efficacy of anti-PD-L1 antibodies (52, 56). In cholangiocarcinoma, an immune mesenchymal subtype has been identified, which is associated with TGFβ signature and poor tumor-infiltrating cells (57). More recently, an elegant study done by Nicolas-Boluda and colleagues (58) investigated the critical determinant of fibrotic tumor progression—the tumor mechanics—and showed that tumor stiffening reversion through collagen crosslinking inhibition improves T cell migration and anti-PD-1 treatment.

Several well-conducted studies in the literature showed that the relation between the immunologic cell in the TME involves complex cellular relationships (25–27). Considering our cohort involving primarily the inflamed tumor-immune phenotype, the characterization of the dendritic cells (DC) would add important information to our study. In fact, antigen-presenting dendritic cells either promote immune attacks by presenting neoantigens to CD8+ T-lymphocytes (conventional DC) or cause immune tolerance by cooperating with regulatory T cells or by inducing an inflammatory environment, which promotes tumor invasion and metastases (plasmocytoid and monocytoid DC) (25). Immune tolerance induced by regulatory T cells anti-inflammatory cytokines normally are related to cancer poor prognostic, as these cells inhibit the action of CD8+ T lymphocytes and NK cells. The plasmocytoid and monocytoid DC induce differentiation of macrophages into the tumor-promoting M2 lineage, which are inducing angiogenesis, promoting tumor growth, invasion, and metastases.

We did not stratify macrophages into M1 (pro-inflammatory; CD68+/IL12+) and M2 (anti-inflammatory; CD68+/IL10+) as previously reported by Popper group (25). It has been shown in cancer that macrophages infiltrate the TME in response to tumor-secreted chemotactic signals and exhibit a tumor-supportive phenotype similar to the M2 phenotype. High macrophage infiltration has been associated with a poor prognosis and increased rates of metastasis in several cancer types, as tumor-associated macrophages can facilitate blood vessel formation to support expanding tumor growth and aid tumor cell intravasation into vasculature. According to Brcic et al. (25) study, alveolar macrophages were predominantly differentiated into tumor-cooperating M2 types in squamous cell and adenocarcinomas of the lung, whereas tumoricidal M1 macrophages were absent or rare. Although we have not evaluated macrophage subtypes, according to the literature, we believe there is a predominance of the M2 subtype in the NSCLC samples analyzed in this study. We also found that Col III was positively correlated with macrophages CD68+ inside of TME, in agreement with Acerbi and colleagues (59) who reported that the parallel alignment of collagen deposition was positively associated with macrophage action. Other reports also stated that tumor-associated macrophages orchestrate the deposition, crosslinking, and linearization of collagen fibers, specifically Col I, Col III, and Col V, at areas of tumor invasiveness (60). Another important cell type in the matricellular are CAFs. Notable, in the current study CAFs were positively correlated with cytotoxic T cell Granzyme B, Col III, and Col V.

Thus, we inferred that collagen-tumor targeting of relevant structural components of the matricellular was activated to drive CAFs to synthetize these fibrillar collagen types creating a bimodal barrier for invasion and metastases (7). The disruption in tissue homeostasis activates matrix fibroblasts into CAFs to synthetize collagen (7), which in turn lead to a fibrotic barrier of tumor which is a major factor in the control and progression of many cancers, including lung cancer, pancreas, breast, and hepatic carcinomas (61–63). In this theatrical stage, fibrillar collagen types are the key players in tumor fibrosis-associated barrier (also called desmoplasia), which is defined as a fibrotic state characterized by an excessive synthesis, deposition, and remodeling of fibrillar collagens surrounding the tumor (64, 65). Collagen represents the most abundant ECM protein, and collagen I and V deposition have been associated with increased desmoplasia leading to increased incidence of tumor formation and metastases (66). Furthermore, CAFs exert anticancer and cancer-induced effects to show bimodal influences for cancer progression (67). Whether it be the increasing action of matricellular stiffening, immune cells, CAFs, and eventual other actors in this theatrical scenario recognize that they are of no meaning without the presence of each other. The relationship between immune cells and mechanosensitive players strengthens the peak of this dramatic piece and remains to bring an ever more fascinating plot that has anything but an ending.

Last but not least, a third important question should be addressed with respect to why only VISTA1 has impact on survival. What about other checkpoints? The survival analysis of our cohort was constructed in two steps. In the first examination, only patients who had pathological stage I, II, and III tumors were included in a stepwise multivariate Cox regression analysis where we added all the immune-matricellular variables that were being assessed. In this model, just two variables were significantly associated with survival time—pathological stage and staining of the tumor for Col III, indicating that integrated with the classic TNM stage classification, Col III can help us to improve risk stratification and patient selection at early stage for anti-fibrotic treatment. In the second examination, we tested the predictive value of the immune-matricellular variables in patients in advanced stage. In this scenario, only VISTA 1 and CD3+lymphocytes, indicating that VISTA 1 integrated with the classic TNM stage and density of CD3 lymphocytes can select patient at advanced stage as candidate for VISTA as another compensatory inhibitory pathway in NSCLC after classical immunotherapy.

In conclusion, the present report suggests that there may be several different phenotypes of inflamed tumor type of immune reaction, and immune excluded type non-inflamed tumors, which may explain different mechanisms for T-cell infiltration and clinical outcome. A better understanding is needed of the mechanical and biochemical barriers to T-cell infiltration, maintenance, and function in the TME. Interruption of these barriers offers the promise of new therapeutic approaches and potential for combined treatments to get more NSCLC responsive to immune and/or collagen therapy.

## Data Availability Statement

The raw data supporting the conclusions of this article will be made available by the authors, without undue reservation.

## Ethics Statement

The studies involving human participants were reviewed and approved by Comissão de Ética para Análise de Projetos de Pesquisa (CAPPesq) do Hospital das Clínicas e da Faculdade de Medicina da Universidade de São Paulo. Written informed consent for participation was not required for this study in accordance with the national legislation and the institutional requirements.

## Author Contributions

Conception and design: VC, LY, and CB. Writing, review, and editing: VC, CB, LY, TP, JR, and PS. Data analysis and interpretation: VC, CB, LY, AV, and WT. Statistical analysis: VC and CB. Provision of study materials or patients: LS, AA, AAb’S, RF, and TT. Administrative support: VC. All authors contributed to the article and approved the submitted version.

## Funding

This work was supported by Sao Paulo Research Foundation (FAPESP; 2018/20403-6), the National Council for Scientific and Technological Development (CNPq; 483005/2012-6), and Coordenação de Aperfeiçoamento de Pessoal de Nível Superior - Brasil (CAPES; Finance Code 001).

## Conflict of Interest

The authors declare that the research was conducted in the absence of any commercial or financial relationships that could be construed as a potential conflict of interest.

## Publisher’s Note

All claims expressed in this article are solely those of the authors and do not necessarily represent those of their affiliated organizations, or those of the publisher, the editors and the reviewers. Any product that may be evaluated in this article, or claim that may be made by its manufacturer, is not guaranteed or endorsed by the publisher.

## References

[1] Herbst RS Morgensztern D Boshoff C. The Biology and Management of Non-Small Cell Lung Cancer. Nature (2018) 553:446–54. 10.1038/nature25183 29364287

[2] American Cancer Society (ACS). Cancer Facts & Figures 2017 [online]. (2017). Available at: https://www.cancer.org/research/cancer-facts-statistics/all-cancer-facts-figures/cancer-facts-figures-2017.html [Accessed April 8, 2021].

[3] Chen Z Fillmore CM Hammerman PS Kim CF Wong KK. Non-Small-Cell Lung Cancers: A Heterogeneous Set of Diseases. Nat Rev Cancer (2014) 14(8):535–46. 10.1038/nrc3775 PMC5712844 25056707

[4] Hanahan D Coussens LM. Accessories to the Crime: Functions of Cells Recruited to the Tumor Microenvironment. Cancer Cell (2012) 21(3):309–22. 10.1016/j.ccr.2012.02.022 22439926

[5] Quail DF Joyce JA. Microenvironmental Regulation of Tumor Progression and Metastasis. Nat Med (2013) 19(11):1423–37. 10.1038/nm.3394 PMC3954707 24202395

[6] Altorki NK Markowitz GJ Gao D Port JL Saxena A Stiles B. The Lung Microenvironment: An Important Regulator of Tumour Growth and Metastasis. Nat Rev Cancer (2019) 19(1):9–31. 10.1038/s41568-018-0081-9 30532012 PMC6749995

[7] Bourgot I Primac I Louis T Noël A Maquoi E. Reciprocal Interplay Between Fibrillar Collagens and Collagen-Binding Integrins: Implications in Cancer Progression and Metastasis. Front Oncol (2020) 10:1488. 10.3389/fonc.2020.01488 33014790 PMC7461916

[8] Kataki A Scheid P Piet M Marie B Martinet N Martinet Y. Tumor Infiltrating Lymphocytes and Macrophages Have a Potential Dual Role in Lung Cancer by Supporting Both Host-Defense and Tumor Progression. J Lab Clin Med (2002) 140(5):320–8. 10.1067/mlc.2002.128317 12434133

[9] Wakabayashi O Yamazaki K Oizumi S Hommura F Kinoshita I Ogura S. CD4+ T Cells in Cancer Stroma, Not CD8+ T Cells in Cancer Cell Nests, Are Associated With Favorable Prognosis in Human Non-Small Cell Lung Cancers. Cancer Sci (2003) 94(11):1003–9. 10.1111/j.1349-7006.2003.tb01392.x PMC11160236 14611679

[10] Ruffini E Asioli S Filosso PL Lyberis P Bruna MC Macrì L. Clinical Significance of Tumor-Infiltrating Lymphocytes in Lung Neoplasms. Ann Thorac Surg (2009) 87(2):365–71 , discussion 371–2. 10.1016/j.athoracsur.2008.10.067 19161739

[11] Hiraoka K Miyamoto M Cho Y Suzuoki M Oshikiri T Nakakubo Y. Concurrent Infiltration by CD8+ T Cells and CD4+ T Cells Is a Favourable Prognostic Factor in Non-Small-Cell Lung Carcinoma. Br J Cancer (2006) 94(2):275–80. 10.1038/sj.bjc.6602934 PMC2361103 16421594

[12] Gooden MJ de Bock GH Leffers N Daemen T Nijman HW. The Prognostic Influence of Tumour-Infiltrating Lymphocytes in Cancer: A Systematic Review With Meta-Analysis. Br J Cancer (2011) 105(1):93–103. 10.1038/bjc.2011.189 21629244 PMC3137407

[13] Blessin NC Simon R Kluth M Fischer K Hube-Magg C Li W. Patterns of TIGIT Expression in Lymphatic Tissue, Inflammation, and Cancer. Dis Markers (2019) 2019:5160565. 10.1155/2019/5160565 30733837 PMC6348838

[14] Horne ZD Jack R Gray ZT Siegfried JM Wilson DO Yousem SA. Increased Levels of Tumor-Infiltrating Lymphocytes Are Associated With Improved Recurrence-Free Survival in Stage 1A Non-Small-Cell Lung Cancer. J Surg Res (2011) 171(1):1–5. 10.1016/j.jss.2011.03.068 21571304

[15] Kilic A Landreneau RJ Luketich JD Pennathur A Schuchert MJ. Density of Tumor-Infiltrating Lymphocytes Correlates With Disease Recurrence and Survival in Patients With Large Non-Small-Cell Lung Cancer Tumors. J Surg Res (2011) 167(2):207–10. 10.1016/j.jss.2009.08.029 19896677

[16] Yoon SM Shaikh T Hallman M. Therapeutic Management Options for Stage III Non-Small Cell Lung Cancer. World J Clin Oncol (2017) 8(1):1–20. 10.5306/wjco.v8.i1.1 28246582 PMC5309711

[17] Jamal-Hanjani M Wilson GA McGranahan N Birkbak NJ Watkins TBK Veeriah S. Tracking the Evolution of Non-Small-Cell Lung Cancer. N Engl J Med (2017) 376:2109–21. 10.1056/NEJMoa1616288 28445112

[18] Goldstraw P Chansky K Crowley J Rami-Porta R Asamura H Eberhardt WE. The IASLC Lung Cancer Staging Project: Proposals for Revision of the TNM Stage Groupings in the Forthcoming (Eighth) Edition of the TNM Classification for Lung Cancer. J Thorac Oncol (2016) 11(1):39–51. 10.1016/j.jtho.2015.09.009 26762738

[19] Travis WD Brambilla E Nicholson AG Yatabe Y Austin JHM Beasley MB. The 2015 World Health Organization Classification of Lung Tumors: Impact of Genetic, Clinical and Radiologic Advances Since the 2004 Classification. J Thorac Oncol (2015) 10(9):1243–60. 10.1097/JTO.0000000000000630 26291008

[20] Guo C Shao R Correa AM Behrens C Johnson FM Raso MG. Prognostic Significance of Combinations of RNA-Dependent Protein Kinase and EphA2 Biomarkers for NSCLC. J Thorac Oncol (2013) 8(3):301–8. 10.1097/JTO.0b013e318282def7 PMC3573252 23370317

[21] Balancin ML Teodoro WR Baldavira CM Prieto TG Farhat C Velosa AP. Different Histological Patterns of Type-V Collagen Levels Confer a Matrices-Privileged Tissue Microenvironment for Invasion in Malignant Tumors With Prognostic Value. Pathol Res Pract (2020) 216(12):153277. 10.1016/j.prp.2020.153277 33223279

[22] Balancin ML Teodoro WR Farhat C de Miranda TJ Assato AK de Souza Silva NA. An Integrative Histopathologic Clustering Model Based on Immuno-Matrix Elements to Predict the Risk of Death in Malignant Mesothelioma. Cancer Med (2020) 9(13):4836–49. 10.1002/cam4.3111 PMC7333849 32391978

[23] Shechter R London A Schwartz M. Orchestrated Leukocyte Recruitment to Immune-Privileged Sites: Absolute Barriers Versus Educational Gates. Nat Rev Immunol (2013) 13(3):206–18. 10.1038/nri3391 23435332

[24] Brodsky AS Xiong J Yang D Schorl C Fenton MA Graves TA. Identification of Stromal Colxα1 and Tumor-Infiltrating Lymphocytes as Putative Predictive Markers of Neoadjuvant Therapy in Estrogen Receptor-Positive/HER2-Positive Breast Cancer. BMC Cancer (2016) 16:274. 10.1186/s12885-016-2302-5 27090210 PMC4835834

[25] Brcic L Stanzer S Krenbek D Gruber-Moesenbacher U Absenger G Quehenberger F. Immune Cell Landscape in Therapy-Naïve Squamous Cell and Adenocarcinomas of the Lung. Virchows Arch (2018) 472(4):589–98. 10.1007/s00428-018-2326-0 PMC5924661 29520483

[26] Cox TR. The Matrix in Cancer. Nat Rev Cancer (2021) 21:217–38. 10.1038/s41568-020-00329-7 33589810

[27] Devaud C Darcy PK Kershaw MH. Foxp3 Expression in T Regulatory Cells and Other Cell Lineages. Cancer Immunol Immunother (2014) 63(9):869–76. 10.1007/s00262-014-1581-4 PMC11028988 25063364

[28] Tao H Mimura Y Aoe K Kobayashi S Yamamoto H Matsuda E. Prognostic Potential of FOXP3 Expression in Non-Small Cell Lung Cancer Cells Combined With Tumor-Infiltrating Regulatory T Cells. Lung Cancer (2012) 75(1):95–101. 10.1016/j.lungcan.2011.06.002 21719142

[29] Coussens LM Pollard JW. Leukocytes in Mammary Development and Cancer. Cold Spring Harb Perspect Biol (2011) 3(3):a003285. 10.1101/cshperspect.a003285 21123394 PMC3039933

[30] Mahajan UM Langhoff E Goni E Costello E Greenhalf W Halloran C. Immune Cell and Stromal Signature Associated With Progression-Free Survival of Patients With Resected Pancreatic Ductal Adenocarcinoma. Gastroenterology (2018) 155(5):1625–39.e2. 10.1053/j.gastro.2018.08.009 30092175

[31] Batista ML Jr Henriques FS Neves RX Olivan MR Matos-Neto EM Alcântara PS. Cachexia-Associated Adipose Tissue Morphological Rearrangement in Gastrointestinal Cancer Patients. J Cachexia Sarcopenia Muscle (2016) 7(1):37–47. 10.1002/jcsm.12037 27066317 PMC4799865

[32] Sharma P Ng C Jana A Padhi A Szymanski P Lee JSH. Aligned Fibers Direct Collective Cell Migration to Engineer Closing and Nonclosing Wound Gaps. Mol Biol Cell (2017) 28:2579–88. 10.1091/mbc.E17-05-0305 PMC5597329 28747440

[33] Ray A Slama ZM Morford RK Madden SA Provenzano PP. Enhanced Directional Migration of Cancer Stem Cells in 3D Aligned Collagen Matrices. Biophys J (2017) 112(5):1023–36. 10.1016/j.bpj.2017.01.007 PMC5355487 28297639

[34] Conklin MW Eickhoff JC Riching KM Pehlke CA Eliceiri KW Provenzano PP. Aligned Collagen Is a Prognostic Signature for Survival in Human Breast Carcinoma. Am J Pathol (2011) 178(3):1221–32. 10.1016/j.ajpath.2010.11.076 PMC3070581 21356373

[35] Kai F Drain AP Weaver VM. The Extracellular Matrix Modulates the Metastatic Journey. Dev Cell (2019) 49(3):332–46. 10.1016/j.devcel.2019.03.026 PMC6527347 31063753

[36] Oudin MJ Weaver VM. Physical and Chemical Gradients in the Tumor Microenvironment Regulate Tumor Cell Invasion, Migration, and Metastasis. Cold Spring Harb Symp Quant Biol (2016) 81:189–205. 10.1101/sqb.2016.81.030817 28424337

[37] Durham NM Nirschl CJ Jackson CM Elias J Kochel CM Anders RA. Lymphocyte Activation Gene 3 (LAG-3) Modulates the Ability of CD4 T-Cells to be Suppressed *In Vivo* . PloS One (2014) 9(11):e109080. 10.1371/journal.pone.0109080 25372844 PMC4220939

[38] Graydon CG Mohideen S Fowke KR. Lag3’s Enigmatic Mechanism of Action. Front Immunol (2021) 11:615317. 10.3389/fimmu.2020.615317 33488626 PMC7820757

[39] Zhou J Yu X Hou L Zhao J Zhou F Chu X. Epidermal Growth Factor Receptor Tyrosine Kinase Inhibitor Remodels Tumor Microenvironment by Upregulating LAG-3 in Advanced Non-Small-Cell Lung Cancer. Lung Cancer (2021) 153:143–9. 10.1016/j.lungcan.2021.01.010 33508527

[40] Champiat S Ileana E Giaccone G Besse B Mountzios G Eggermont A. Incorporating Immune-Checkpoint Inhibitors Into Systemic Therapy of NSCLC. J Thorac Oncol (2014) 9(2):144–53. 10.1097/JTO.0000000000000074 24419410

[41] Willimsky G Blankenstein T. Sporadic Immunogenic Tumours Avoid Destruction by Inducing T-Cell Tolerance. Nature (2005) 437(7055):141–6. 10.1038/nature03954 16136144

[42] DeLong P Carroll RG Henry AC Tanaka T Ahmad S Leibowitz MS. Regulatory T Cells and Cytokines in Malignant Pleural Effusions Secondary to Mesothelioma and Carcinoma. Cancer Biol Ther (2005) 4(3):342–6. 10.4161/cbt.4.3.1644 15846066

[43] Sakaguchi S. Naturally Arising CD4+ Regulatory T Cells for Immunologic Self-Tolerance and Negative Control of Immune Responses. Annu Rev Immunol (2004) 22:531–62. 10.1146/annurev.immunol.21.120601.141122 15032588

[44] Xia M Zhao MQ Wu K Lin XY Liu Y Qin YJ. Investigations on the Clinical Significance of FOXP3 Protein Expression in Cervical Oesophageal Cancer and the Number of FOXP3+ Tumour-Infiltrating Lymphocytes. J Int Med Res (2013) 41(4):1002–8. 10.1177/0300060513488504 23760912

[45] Hao Q Li W Zhang C Qin X Xue X Li M. Tnfα Induced FOXP3-Nfκb Interaction Dampens the Tumor Suppressor Role of FOXP3 in Gastric Cancer Cells. Biochem Biophys Res Commun (2013) 430(1):436–41. 10.1016/j.bbrc.2012.11.039 23178569

[46] Yoshii M Tanaka H Ohira M Muguruma K Iwauchi T Lee T. Expression of Forkhead Box P3 in Tumour Cells Causes Immunoregulatory Function of Signet Ring Cell Carcinoma of the Stomach. Br J Cancer (2012) 106(10):1668–74. 10.1038/bjc.2012.141 PMC3349176 22569001

[47] Lal A Chan L Devries S Chin K Scott GK Benz CC. FOXP3-Positive Regulatory T Lymphocytes and Epithelial FOXP3 Expression in Synchronous Normal, Ductal Carcinoma *In Situ*, and Invasive Cancer of the Breast. Breast Cancer Res Treat (2013) 139(2):381–90. 10.1007/s10549-013-2556-4 23712790

[48] Kuczek DE Larsen AMH Thorseth ML Carretta M Kalvisa A Siersbæk MS. Collagen Density Regulates the Activity of Tumor-Infiltrating T Cells. J Immunother Cancer (2019) 7(1):68. 10.1186/s40425-019-0556-6 30867051 PMC6417085

[49] Bougherara H Mansuet-Lupo A Alifano M Ngô C Damotte D Le Frère-Belda MA. Real-Time Imaging of Resident T Cells in Human Lung and Ovarian Carcinomas Reveals How Different Tumor Microenvironments Control T Lymphocyte Migration. Front Immunol (2015) 6:500. 10.3389/fimmu.2015.00500 26528284 PMC4600956

[50] Salmon H Franciszkiewicz K Damotte D Dieu-Nosjean MC Validire P Trautmann A. Matrix Architecture Defines the Preferential Localization and Migration of T Cells Into the Stroma of Human Lung Tumors. J Clin Invest (2012) 122(3):899–910. 10.1172/JCI45817 22293174 PMC3287213

[51] Carstens JL Correa de Sampaio P Yang D Barua S Wang H Rao A. Spatial Computation of Intratumoral T Cells Correlates With Survival of Patients With Pancreatic Cancer. Nat Commun (2017) 8:15095. 10.1038/ncomms15095 28447602 PMC5414182

[52] Mariathasan S Turley SJ Nickles D Castiglioni A Yuen K Wang Y. Tgfβ Attenuates Tumour Response to PD-L1 Blockade by Contributing to Exclusion of T Cells. Nature (2018) 554(7693):544–8. 10.1038/nature25501 PMC6028240 29443960

[53] Budhu S Schaer DA Li Y Toledo-Crow R Panageas K Yang X. Blockade of Surface-Bound TGF-β on Regulatory T Cells Abrogates Suppression of Effector T Cell Function in the Tumor Microenvironment. Sci Signal (2017) 10(494):eaak9702. 10.1126/scisignal.aak9702 28851824 PMC5851440

[54] Herbst RS Soria JC Kowanetz M Fine GD Hamid O Gordon MS. Predictive Correlates of Response to the Anti-PD-L1 Antibody MPDL3280A in Cancer Patients. Nature (2014) 515(7528):563–7. 10.1038/nature14011 PMC4836193 25428504

[55] Turley SJ Cremasco V Astarita JL. Immunological Hallmarks of Stromal Cells in the Tumour Microenvironment. Nat Rev Immunol (2015) 15(11):669–82. 10.1038/nri3902 26471778

[56] Tauriello DVF Palomo-Ponce S Stork D Berenguer-Llergo A Badia-Ramentol J Iglesias M. Tgfβ Drives Immune Evasion in Genetically Reconstituted Colon Cancer Metastasis. Nature (2018) 554(7693):538–43. 10.1038/nature25492 29443964

[57] Job S Rapoud D Dos Santos A Gonzalez P Desterke C Pascal G. Identification of Four Immune Subtypes Characterized by Distinct Composition and Functions of Tumor Microenvironment in Intrahepatic Cholangiocarcinoma. Hepatology (2020) 72(3):965–81. 10.1002/hep.31092 PMC7589418 31875970

[58] Nicolas-Boluda A Vaquero J Vimeux L Guilbert T Barrin S Kantari-Mimoun C. Tumor Stiffening Reversion Through Collagen Crosslinking Inhibition Improves T Cell Migration and Anti-PD-1 Treatment. Elife (2021) 10:e58688. 10.7554/eLife.58688 34106045 PMC8203293

[59] Acerbi I Cassereau L Dean I Shi Q Au A Park C. Human Breast Cancer Invasion and Aggression Correlates With ECM Stiffening and Immune Cell Infiltration. Integr Biol (Camb) (2015) 7(10):1120–34. 10.1039/c5ib00040h PMC4593730 25959051

[60] Afik R Zigmond E Vugman M Klepfish M Shimshoni E Pasmanik-Chor M. Tumor Macrophages Are Pivotal Constructors of Tumor Collagenous Matrix. J Exp Med (2016) 213(11):2315–31. 10.1084/jem.20151193 PMC5068227 27697834

[61] Bissell MJ Hines WC. Why Don’t We Get More Cancer? A Proposed Role of the Microenvironment in Restraining Cancer Progression. Nat Med (2011) 17(3):320–9. 10.1038/nm.2328 PMC3569482 21383745

[62] Pietras K Ostman A. Hallmarks of Cancer: Interactions With the Tumor Stroma. Exp Cell Res (2010) 316(8):1324–31. 10.1016/j.yexcr.2010.02.045 20211171

[63] Liu W Wei H Gao Z Chen G Liu Y Gao X. COL5A1 may Contribute the Metastasis of Lung Adenocarcinoma. Gene (2018) 665:57–66. 10.1016/j.gene.2018.04.066 29702185

[64] Venning FA Wullkopf L Erler JT. Targeting ECM Disrupts Cancer Progression. Front Oncol (2015) 5:224. 10.3389/fonc.2015.00224 26539408 PMC4611145

[65] Pankova D Chen Y Terajima M Schliekelman MJ Baird BN Fahrenholtz M. Cancer-Associated Fibroblasts Induce a Collagen Cross-Link Switch in Tumor Stroma. Mol Cancer Res (2016) 14(3):287–95. 10.1158/1541-7786.MCR-15-0307 PMC4794404 26631572

[66] Gilkes DM Semenza GL Wirtz D. Hypoxia and the Extracellular Matrix: Drivers of Tumour Metastasis. Nat Rev Cancer (2014) 14(6):430–9. 10.1038/nrc3726 PMC4283800 24827502

[67] Gieniec KA Butler LM Worthley DL Woods SL. Cancer-Associated Fibroblasts-Heroes or Villains? Br J Cancer (2019) 121(4):293–302. 10.1038/s41416-019-0509-3 31289350 PMC6738083

