# Supplementary material for: In Situ Overexpression of Matricellular Mechanical Proteins Demands Functional Immune Signature and Mitigates Non-Small Cell Lung Cancer Progression
Source: Front Immunol. 2021 Aug 16;12:714230. doi: 10.3389/fimmu.2021.714230 (PMC8415570; doi:10.3389/fimmu.2021.714230)
Supplement: Supplementary file 1 [file Table_1.docx]

**Supplementary Table 1.** Expression of immune and matricellular proteins in NSCLC

| **Immune and matricellular-proteins** | **Mean** | **Median** | **IQR** |
| --- | --- | --- | --- |
|  | **(cell/mm² or fibers/mm²)** | | |
| T cells CD3+ ^a^ | 485.34 | 278.50 | 504.99 |
| Cytotoxic T cells CD8^+a^ | 480.27 | 217.38 | 417.23 |
| Cytotoxic T cells Granzyme B | 25.44 | 6.19 | 19.50 |
| Malignant cells PD-L1 | 45.97 | 0.37 | 2.84 |
| TILs LAG-3 | 278.97 | 95.33 | 308.70 |
| TILs CTLA-4+ | 245.12 | 227.50 | 186.39 |
| Macrophages CD68+ | 225.24 | 225.78 | 107.62 |
| Natural-killer T cells CD57+ | 153.32 | 116.55 | 149.42 |
| Regulatory T cells CD4+ | 269.88 | 98.32 | 310.50 |
| Regulatory T cells FOXP3+ | 170.36 | 127.00 | 109.47 |
| B Lymphocytes CD20+ | 447.45 | 154.18 | 359.69 |
| TILs VISTA 1 | 116.96 | 52.86 | 144.85 |
| Col I | 2.81 | 2.54 | 2.71 |
| Col III | 25.04 | 24.89 | 11.15 |
| Col V | 14.42 | 13.18 | 8.20 |
| CAFs | 13.33 | 12.39 | 7.23 |

^a^ Some cases had missing information: CD3+ (46); CD8^+^ (1); PD-L1 (20); CTLA-4+ (1)

Abbreviations: IQR, interquartile range; TILs, tumor infiltrating lymphocytes; PD-L1, programmed death ligand 1; LAG-3, lymphocyte activating gene 3; CTLA-4, Cytotoxic T-Lymphocyte Associated Protein 4; FOXP3, Fork head box protein P3; VISTA 1, V-domain Ig suppressor of T cell activation; Col I, collagen type I; Col III, collagen type III; Col V, collagen type V; CAFs, cancer-associated fibroblasts.
