# Supplementary material for: In Situ Overexpression of Matricellular Mechanical Proteins Demands Functional Immune Signature and Mitigates Non-Small Cell Lung Cancer Progression
Source: Front Immunol. 2021 Aug 16;12:714230. doi: 10.3389/fimmu.2021.714230 (PMC8415570; doi:10.3389/fimmu.2021.714230)
Supplement: Supplementary file 2 [file Table_2.docx]

**Supplementary Table 2.** Quantitative distribution of immune-matricellular-associated proteins of the different histotypes (N=120; Kruskal-Wallis; P<0.05)

| **Immune and matricellular-proteins** |  | **Histologic subtypes** | |
| --- | --- | --- | --- |
|  |  | **X²(2)** | ***P-*value** |
| T cells CD3+ ^a^ |  | 1.827 | 0.401 |
| Cytotoxic T cells CD8^+a^ |  | 6.777 | **0.034** |
| Cytotoxic T cells Granzyme B |  | 1.320 | 0.517 |
| Malignant cells PD-L1 |  | 0.774 | 0.679 |
| TILs LAG-3 |  | 1.764 | 0.414 |
| TILs CTLA-4+ |  | 8.827 | **0.012** |
| Macrophages CD68+ |  | 16.29 | **<0.001** |
| Natural-killer T cells CD57+ |  | 5.278 | 0.071 |
| Regulatory T cells CD4+ |  | 3.607 | 0.165 |
| Regulatory T cells FOXP3+ |  | 4.528 | 0.104 |
| B Lymphocytes CD20+ |  | 0.941 | 0.625 |
| TILs VISTA 1 |  | 1.019 | 0.601 |
| Col I |  | 0.918 | 0.632 |
| Col III |  | 1.634 | 0.442 |
| Col V |  | 0.574 | 0.750 |
| CAFs |  | 0.249 | 0.883 |

^a^ Some cases had missing information: CD3+ (46); CD8^+^ (1); PD-L1 (20); CTLA-4+ (1)

Abbreviations: TILs, tumor infiltrating lymphocytes; PD-L1, programmed death ligand 1; LAG-3, lymphocyte activating gene 3; CTLA-4, Cytotoxic T-Lymphocyte Associated Protein 4; FOXP3, Fork head box protein P3; VISTA 1, V-domain Ig suppressor of T cell activation; Col I, collagen type I; Col III, collagen type III; Col V, collagen type V; CAFs, cancer-associated fibroblasts; X^2^(2), Chi-square(degree of freedom).

Bolded values refer to a P-value with statistical significance (P<0.05).
