# Supplementary material for: In Situ Overexpression of Matricellular Mechanical Proteins Demands Functional Immune Signature and Mitigates Non-Small Cell Lung Cancer Progression
Source: Front Immunol. 2021 Aug 16;12:714230. doi: 10.3389/fimmu.2021.714230 (PMC8415570; doi:10.3389/fimmu.2021.714230)
Supplement: Supplementary file 3 [file Table_3.docx]

**Supplementary Table 3.** Univariate analysis using a Cox proportional hazards model.

| **Characteristic** | **Univariate Analysis^b^** | | |
| --- | --- | --- | --- |
|  | **HR (95% CI)** | **HR** | ***P* value** |
| **Gender** |  |  |  |
| Male | 1.788 (1.064-3.004) | 0.581 | **0.028** |
| Female (reference) |  |  |  |
| **Age (years)** |  |  |  |
| ≤ 65 | 1.511 (0.915-2.496) | 0.413 | 0.107 |
| > 65 (reference) |  |  |  |
| **Smoker Status^a^** |  |  |  |
| Non-Smoker | 0.427 (0.167-1.094) | -0.850 | 0.076 |
| Smoker/Ex-smoker (reference) |  |  |  |
| **Histological type** |  |  |  |
| Adenocarcinoma | 0.738 (0.262-2.082) | -0.304 | 0.566 |
| Squamous cell carcinoma | 1.416 (0.492-4.077) | 0.348 | 0.519 |
| Large cell carcinoma (reference) |  |  | **0.048** |
| **Clinical stage†** |  |  |  |
| I+II | 0.394 (0.240-0.649) | -0.930 | **0.000** |
| III+IV (reference) |  |  |  |
| **T stage†** |  |  |  |
| T1/T2 | 0.440 (0.258-0.749) | -0.822 | **0.003** |
| T3/T4 (reference) |  |  |  |
| **N stage†** |  |  |  |
| N0 | 0.545 (0.330-0.899) | -0.607 | **0.017** |
| N1/N2 (reference) |  |  |  |
| **M stage^a^†** |  |  |  |
| M0 | 0.241 (0.141-0.411) | -1.422 | **0.000** |
| M1 (reference) |  |  |  |
| **Adjuvant therapy** |  |  |  |
| **Chemotherapy** |  |  |  |
| No | 0.567 (0.342-0.940) | -0.567 | **0.028** |
| Yes (reference) |  |  |  |
| **Radiotherapy** |  |  |  |
| No | 0.427 (0.258-0.709) | -0.850 | **0.001** |
| Yes (reference) |  |  |  |
| **Protein expression** (≤ median vs > median) |  |  |  |
| **T cells CD3+** |  |  |  |
| ≤ 278.50 | 2.116 (1.032-4.339) | 0.750 | **0.041** |
| > 278.50 (reference) |  |  |  |
| **Cytotoxic T cells CD8^+a^** |  |  |  |
| ≤ 217.38 | 1.290 (0.782-2.128) | 0.255 | 0.319 |
| > 217.38 (reference) |  |  |  |
| **Cytotoxic T cells Granzyme B** |  |  |  |
| ≤ 6.19 | 1.604 (0.973-2.644) | 0.473 | 0.064 |
| > 6.19 (reference) |  |  |  |
| **Malignant cells PD-L1^a^** |  |  |  |
| ≤ 0.37 | 1.127 (0.663-1.918) | 0.120 | 0.658 |
| > 0.37 (reference) |  |  |  |
| **TILs LAG-3** |  |  |  |
| ≤ 95.33 | 1.060 (0.645-1.742) | 0.059 | 0.817 |
| > 95.33 (reference) |  |  |  |
| **TILs CTLA-4+^a^** |  |  |  |
| ≤ 8200.67 | 1.094 (0.664-1.801) | 0.090 | 0.725 |
| > 8200.67 (reference) |  |  |  |
| **Macrophages CD68+** |  |  |  |
| ≤ 7383.68 | 1.071 (0.653-1.757) | 0.068 | 0.786 |
| > 7383.68 (reference) |  |  |  |
| **Natural killers cells CD57+** |  |  |  |
| ≤ 4496.65 | 0.712 (0.433-1.171) | -0.340 | 0.181 |
| > 4496.65 (reference) |  |  |  |
| **Regulatory T cells CD4+** |  |  |  |
| ≤ 98.32 | 1.737 (1.050-2.873) | 0.552 | **0.031** |
| > 98.32 (reference) |  |  |  |
| **Regulatory T cells FOXP3+** |  |  |  |
| ≤ 5201.72 | 1.092 (0.665-1.792) | 0.088 | 0.728 |
| > 5201.72 (reference) |  |  |  |
| **B Lymphocytes CD20+** |  |  |  |
| ≤ 154.19 | 0.799 (0.485-1.314) | -0.225 | 0.377 |
| > 154.19 (reference) |  |  |  |
| **TILs VISTA 1** |  |  |  |
| ≤ 52.86 | 0.626 (0.377-1.040) | -0.468 | 0.071 |
| > 52.86 (reference) |  |  |  |
| **COL I^a^** |  |  |  |
| ≤ 2.87 | 1.373 (0.833-2.262) | 0.317 | 0.213 |
| > 2.87 (reference) |  |  |  |
| **COL III^a^** |  |  |  |
| ≤ 31.00 | 1.453 (0.883-2.391) | 0.373 | 0.142 |
| > 31.00 (reference) |  |  |  |
| **COL V^a^** |  |  |  |
| ≤ 14.89 | 1.322 (0.802-2.180) | 0.279 | 0.273 |
| > 14.89 (reference) |  |  |  |
| **CAFs^a^** |  |  |  |
| ≤ 13.01 | 1.354 (0.819-2.239) | 0.303 | 0.238 |
| > 13.01 (reference) |  |  |  |

Abbreviations: HR= hazard ratio (β coefficient); CI=confidence interval; TILs, tumor infiltrating lymphocytes; PD-L1, programmed death ligand 1; LAG-3, lymphocyte activating gene 3; CTLA-4, Cytotoxic T-Lymphocyte Associated Protein 4; FOXP3, Fork head box protein P3; VISTA 1, V-domain Ig suppressor of T cell activation; Col I, collagen type I; Col III, collagen type III; Col V, collagen type V; CAFs, cancer-associated fibroblasts.

^a^ Some cases had missing follow-up information: smoke status (31); M stage (10); CD8^+^ (1); CTLA-4+ (1); PD-L1 (21); CD3+ (46); COL I (71); COL III (71); COL V (71); CAFs (71).

^b^ Univariate analysis was carried out without any adjustment in order to generate hazard ratios with confidence intervals for individual risk for each of the parameters on survival.

† 8th Edition International Association for the Study of Lung Cancer (18)

Bolded values refer to a P-value with statistical significance (P<0.05).
